# The Third Intron of the Interferon Regulatory Factor-8 Is an Initiator of Repressed Chromatin Restricting Its Expression in Non-Immune Cells

**DOI:** 10.1371/journal.pone.0156812

**Published:** 2016-06-03

**Authors:** Mamduh Khateb, Nitsan Fourier, Ofer Barnea-Yizhar, Sigal Ram, Ekaterina Kovalev, Aviva Azriel, Ulfert Rand, Manabu Nakayama, Hansjörg Hauser, Lior Gepstein, Ben-Zion Levi

**Affiliations:** 1 Department of Biotechnology and Food Engineering, Technion—Israel Institute of Technology, Haifa, Israel; 2 Rappaport Faculty of Medicine and Research Institute, Technion—Israel Institute of Technology, Haifa, Israel; 3 Department of Gene Regulation and Differentiation, Helmholtz Centre for Infection Research, Braunschweig, Germany; 4 Department of Technology Development, Kazusa DNA Research Institute, Kazusa-Kamatari, Kazusa, Japan; Augusta University, UNITED STATES

## Abstract

Interferon Regulatory Factor-8 (IRF-8) serves as a key factor in the hierarchical differentiation towards monocyte/dendritic cell lineages. While much insight has been accumulated into the mechanisms essential for its hematopoietic specific expression, the mode of restricting IRF-8 expression in non-hematopoietic cells is still unknown. Here we show that the repression of IRF-8 expression in restrictive cells is mediated by its 3^rd^ intron. Removal of this intron alleviates the repression of Bacterial Artificial Chromosome (BAC) IRF-8 reporter gene in these cells. Fine deletion analysis points to conserved regions within this intron mediating its restricted expression. Further, the intron alone selectively initiates gene silencing only in expression-restrictive cells. Characterization of this intron’s properties points to its role as an initiator of sustainable gene silencing inducing chromatin condensation with suppressive histone modifications. This intronic element cannot silence episomal transgene expression underlining a strict chromatin-dependent silencing mechanism. We validated this chromatin-state specificity of IRF-8 intron upon *in-vitro* differentiation of induced pluripotent stem cells (iPSCs) into cardiomyocytes. Taken together, the IRF-8 3^rd^ intron is sufficient and necessary to initiate gene silencing in non-hematopoietic cells, highlighting its role as a nucleation core for repressed chromatin during differentiation.

## Introduction

Bone marrow derived Hematopoietic Stem Cells (HSC) give rise to lineage specific progenitors among which is the Common Myeloid Progenitor (CMP) cells that can further differentiate to Granulocyte/Monocyte Progenitors (GMP). The latter is the source for three subsets of myeloid cells: granulocytes, monocytes and dendritic cells (DCs). Transcription factors play key roles in this differentiation process through the regulation of a characteristic set of lineage-specific target genes [1–4].

Interferon Regulatory Factor -8 (IRF-8) is a nuclear transcription factor that belongs to the IRF family and is constitutively expressed in the hematopoietic lineages of monocyte/macrophage cells, DCs, B-cells and at low levels in resting T-cells [5, 6]. IRF-8 serves as a key factor in the hierarchical differentiation from HSC towards the monocyte/DC linages. Expression of IRF-8 can be further induced in these cells by IFN-γ [7]. Mice with IRF-8 null mutation are defective in the ability of myeloid progenitor cells to mature towards monocyte/DC lineages. These KO mice eventually develop chronic myelogenous leukemia (CML) like syndrome [8]. Together, these observations highlight the role for IRF-8 in monopoiesis and as a tumor suppressor gene of myelo-leukemias such as CML.

In an attempt to identify the molecular mechanisms leading to this lineage restricted expression of IRF-8, we employed IRF-8 Bacterial Artificial Chromosome (BAC) reporter constructs. Such BAC constructs harbor the regulatory regions as well as the *cis* and distal elements that define expression domains of a gene of interest such as scaffold/matrix attachment regions that isolate the gene from distal regulation [9]. Using successive deletion strategy, we demonstrate that the 3^rd^ intron of IRF-8 harbors regulatory elements that suppress its expression in restrictive cells. We provide evidence showing that changes in chromatin architecture, e.g. nucleosome occupancy and histone post-translational modifications (PTM) profile, are mediators of active suppression of IRF-8 expression in restrictive cells. Cloning of IRF-8 3^rd^ intron near a reporter gene in a retroviral vector results in gene silencing only in restrictive cells, pointing to its role as nucleation core for chromatin condensation when the viral DNA assembles chromatin conformation upon integration. Interestingly, this intronic element is not engaged in repressed chromatin activity in iPSCs, harboring chromatin in a naïve state [10]. However, significant repression of this reporter gene construct is elicited by this intronic element when these cells differentiate into cardiomyocytes that are restrictive for IRF-8 expression. Thus, our results point to a novel activity of an intronic element that acts as an organizer of repressed chromatin state in expression restrictive cells.

## Materials and Methods

### Cell lines

NIH3T3 (Mouse embryo fibroblast), RAW (RAW267.4, Murine monocytes/macrophages-like) and 293FT (Human embryonal kidney) were obtained from ATCC, Manassas, Virginia, USA (CRL-1658, TIB-71 and CRL-3216, respectively). These cell lines were maintained in DMEM supplemented with 10% FCS, 2.5 μg/ml Amphotericin and 50 μg/ml Gentamycin Sulfate (Biological Industries, Beit-Haemek, Israel). Mouse iPS cell line (miPS-B6-GFP) was provided by Prof. Lior Gepstein. Undifferentiated colonies were cultured on mitotically inactivated mouse embryonic fibroblasts (MEF) feeder layer, as previously described [11]. Cells were maintained in DMEM supplemented with 15% FCS (Biological Industries), 0.1% leukemia inhibitory factor (LIF) (Millipore), 1mM L-glutamine, 0.1mM Mercaptoethanol, and 1% nonessential amino acid stock (all from Invitrogen).

### Animals

C57BL/6J (Harlan Biotech, Rehovot, Israel) mice were maintained in microisolator cages in a viral pathogen-free facility. All animal studies and experimental protocols were approved by the Animal Care and Use Committee of the Technion (Ethics number: IL-104-09-13). Prior to cell collection mice were euthanized by CO_2_ asphyxiation by trained personnel and all efforts were made to minimize suffering.

### Cell preparation and culture of BMDM and GMP

Bone Marrow Derived Macrophages (BMDMs)–Bone marrow (BM) cells were isolated from femurs and tibias of 6–8 weeks old C57BL/6J females and cultured in DMEM supplemented with 30% CCL1 cell culture supernatant (source for M-CSF), 20% FCS, 2.5 μg/ml Amphotericin and 50 μg/ml Gentamycin Sulfate. After 7 days of cultivation, typical BMDMs were obtained (adherent cells).

GMPs—Bone marrow cells were isolated as described above and grown in DMEM supplemented with 10% FCS, 10% filtered WEHI cell culture supernatant (a source for IL-3), 10 ng/ml recombinant mouse stem cell factor (rmSCF) (Peprotech, Rocky Hill, NJ, USA), 2.5 μg/ml Amphotericin and 50 μg/ml Gentamycin Sulfate. After 7 days of cultivation, non-adherent cells were collected. BMDM and GMP cells phenotype was verified by flow cytometry with anti-CD34 antibody and quantitative real time PCR (qRT-PCR) with specific primers targeting cell type-specific genes, such as CD34, Tie2 (GMP) and M-CSF receptor (BMDM) (S1 Table). Characterization of mouse BM derived GMP and BMDM cells is detailed in S1 Fig. 90% of the progenitor cell population was CD34^high^ (S1A Fig) and exhibited high mRNA levels of the progenitors-associated genes CD34 and Tie2 (S1B and S1C Fig, respectively) compared to mature BMDM. On the other hand, BMDM exhibited relatively high expression level of the macrophage-associated gene, M-CSF receptor (S1D Fig). In contrast to BMDM, GMPs were restrictive for IRF-8 expression and were not responsive to IFN-γ induction (S1E Fig).

### BAC IRF-8 reporter constructs

The BAC clone 7H10 was obtained from the BACPAC Resource Center, Children's Hospital Oakland Research Institute, Oakland, California, USA. This BAC clone harbors 219,907bp encompassing the murine IRF-8 locus as described hereafter. Construction of the various BAC-IRF-8 reporter constructs was generated using the Red ET cloning procedures [12] as outlined in the text. In principal, a reporter cassette containing a reporter gene open reading frame followed by Neo resistance gene placed under the control of dual promoters, phosphoglycerate kinase promoter (PGK) and SV40 early enhancer/promoter region, conferring Neo resistance in *E*.*coli* and mammalian cells, respectively, was cloned to pSK plasmid. The reporter cassette was amplified by PCR with two primers harboring 50bp homologous arms to the site of integration. Homologous recombination was performed by the recombination proteins of bacteriophage lambda (ET recombination) [12]. The exact integration of the reporter cassette for each BAC IRF-8 construct was verified by DNA sequencing.

### Generating BAC IRF-8 reporter construct stable clones

**Fig 1:**
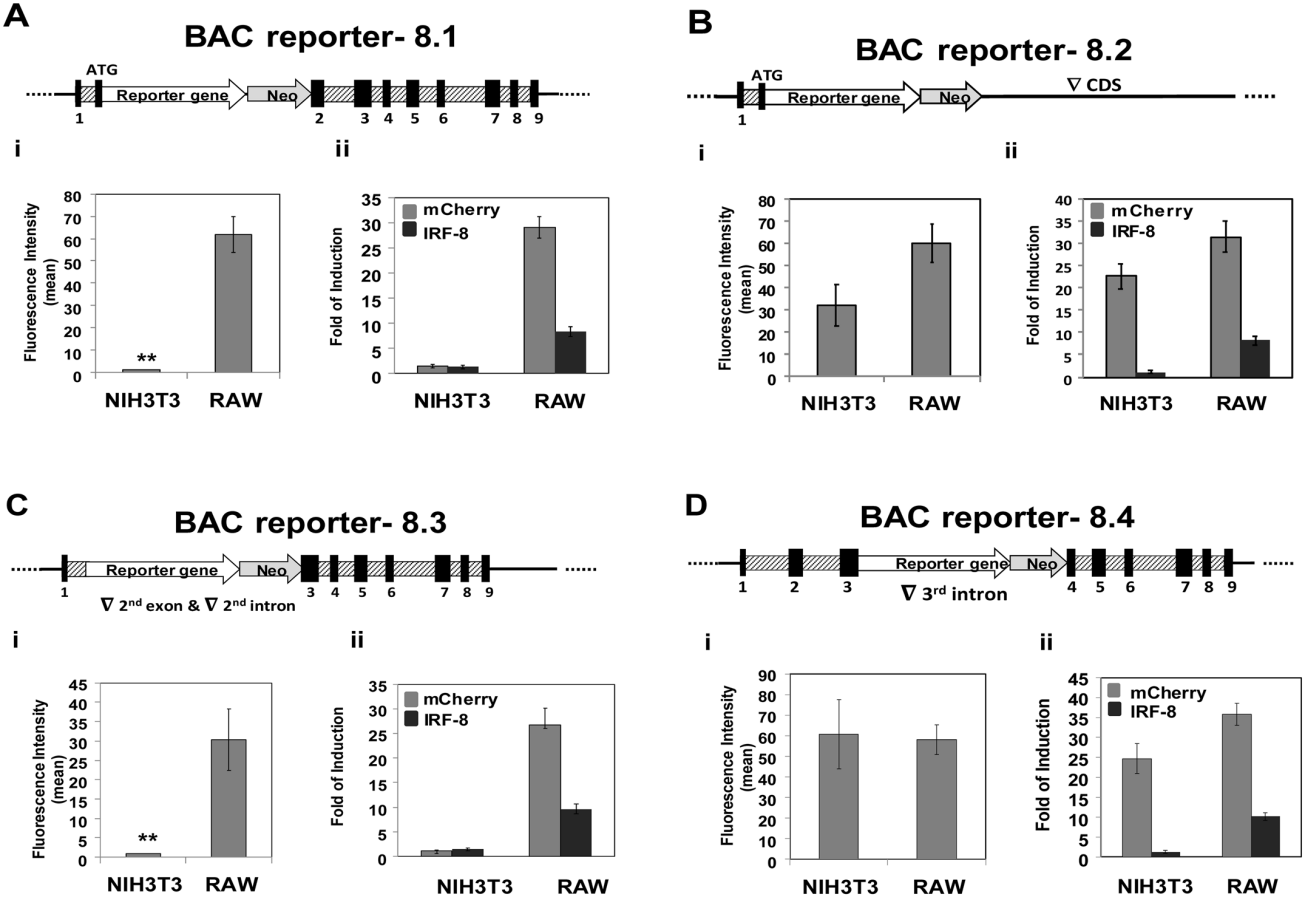
Deletion analysis using BAC-IRF-8 reporter constructs points to the role of the 3^rd^ intron in modulating lineage specific expression of IRF-8. Schematic illustrations of the various BAC-IRF-8 constructs, to which a cassette containing a fluorescent reporter (mCherry) and a selectable marker (Neo driven by the PKG promoter) was inserted, are shown. (**A**) BAC-IRF-8.1- the cassette was inserted to the first ATG. (**B**) BAC-IRF-8.2- the cassette was swapped with the entire IRF-8 coding region (CDS). (**C**) The reporter construct BAC-IRF-8.3 is similar to that illustrated in panel A except that the 2^nd^ intron was deleted. (**D**) The reporter construct BAC-IRF-8.4 is similar to that illustrated in panel A except that the 3^rd^ intron was deleted. Exons (black boxes) are numbered. RAW and NIH3T3 cells were transfected with the various BAC constructs and the fluorescence intensity of the reporter gene in three different RAW and NIH3T3 stable clones, harboring 1–2 copies of the BAC reporter construct, was visualized under fluorescent microscope and quantified as described under Materials and Methods. (Students t-test,** p<0.01). Additionally, RNA was extracted from 3 independent clones for each BAC construct before and following treatment with IFN-γ and the Fold of Induction levels of the reporter gene and the endogenous IRF-8 were determined by real-time qRT-PCR (ii section of each panel).

7*10^5^ cells of either RAW or NIH3T3 cells were seeded in 6 wells tissue culture plates with 2 ml of fresh DMEM medium containing 5% FCS. 18 hrs later, cells were transfected with 6 μg of the DNA corresponding to the various BAC IRF-8 reporter constructs using Metafectene Pro according to the manufacturer's protocol (Biontex laboratories GmbH, Germany). Cells were harvested 24 hrs later and plated on 10 cm culture dishes and after additional 16 hrs, Geneticin (G418) was added to select for stably transfected clones. Individual clones were isolated about 14–18 days later, and the copy number of various transfected BAC IRF-8 reporter constructs was determined by qPCR of isolated genomic DNA in comparison to endogenous single copy genes such as PML and Nramp1. Additionally, PCR using genomic DNA as template was performed with several primer sets spanning along the BAC IRF-8 reporter construct to verify that the whole BAC IRF-8 reporter construct was integrated. Primer sets are detailed in S1 Table. The basal expression level of IRF-8 in the expression permissive RAW cell line is relatively low. To augment its expression level to be easily detected under fluorescent microscope, we treated the cells with IFN-γIRF-8 restrictive cells, such as NIH3T3 cell line, GMP cells, and miPSC cells do not respond to IFN-γ (Figs 1Aii, S1D and S5, respectively).

### Deletions using VCre recombination system

**Fig 2:**
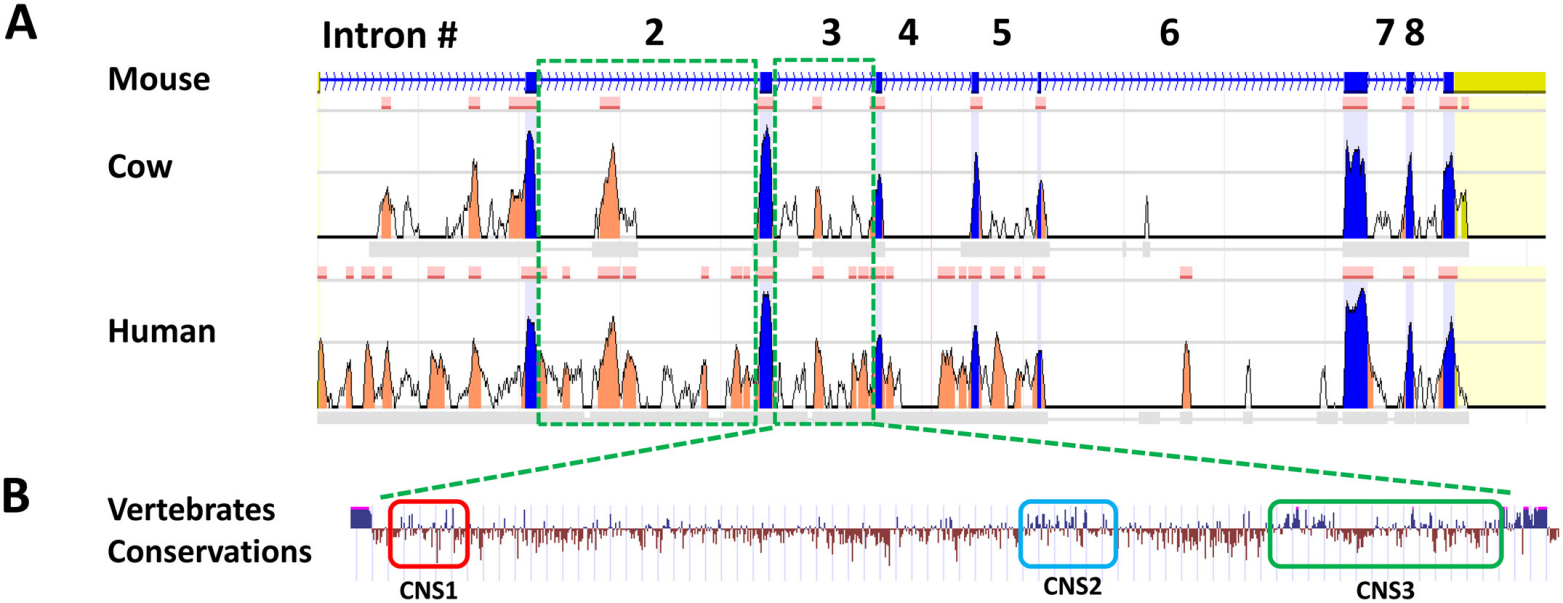
Bioinformatics analysis of the IRF-8 coding region with special detail on the 3^rd^ intron. The upper panel illustrates comparative sequence analysis of IRF-8 coding region using ECR Browser [23]. Conserved sequences (sequences that are longer than 100bp and have at least of 70% identity) in the IRF-8 coding region between mouse, cow and human genomes are aligned. Exons are indicated in blue and conserved non-coding sequences (CNS) within the introns indicated in orange. The 2^nd^ and the 3^rd^ intron are boxed by dotted lines. Detailed illustration of the 3^rd^ intron (1730bp) is presented at the lower panel revealing three conserved regions between mouse (mm10 dataset) and human (hg19 dataset), designated CNS 1, 2 and 3 (boxed) [53].

Since our BAC constructs harbor both classical Cre/loxP sites as well as FLP-FRT sites [13], we turned to a new site-specific recombination system, VCre/VLoxP [14]. Using a recombineering approach as described above, new BAC constructs, in which the IRF-8 3^rd^ intron or three evolutionary Conserved Non-coding Sequences (CNS, Fig 2) were flanked by two VLoxP sites, were generated by PCR. VCre recombinase was sub-cloned from pTurboVCre [14] to pMSCV-Puromycin (Puro) retroviral vector. The VCre constructs were transfected to NIH3T3 cells and numerous cell clones were collected. To perform 3^rd^ intron deletion within the cells, clones were transduced with either empty retroviral vector or retroviral vector encoding for the VCre gene. Genomic DNA was extracted from each transduced clone and VCre efficiency was analyzed using real time qPCR with primer pair flanking the 3^rd^ intron, primer pair from within the 3^rd^ intron and primer pair targeting tGFP for control (Del int3, IRF-8 int3 amplicon8, and tGFP, S1 Table, Respectively). All clones exhibited at least 70% deletion efficiency (data not shown). Subsequently, the reporter gene expression was analyzed.

### Fluorescent cell microscopy

Fluorescent NIH3T3 and RAW cells were visualized under the exact same conditions using Inverted Cell Observer (Axiovert 200, Zeiss). Fluorescence intensity was quantified using AxioVision software (Zeiss).

### Formaldehyde Assisted Isolation of Regulatory Elements (FAIRE)

The protocol was adopted from Simon and Giresi [15]. Briefly, cells were grown to 80–90% confluence, harvested, cross-linked with 1% formaldehyde (5 min for RAW cells and 15 min for NIH3T3 cells) and lysed. Genomic DNA was sheared using VibraCell VCX750 micro-tip sonicator (Sonics & Materials, Inc.) and chromatin was divided into INPUT and FAIRE DNA samples. FAIRE DNA samples were subjected to phenol/chloroform extraction; the nucleosome depleted DNA phase (aqueous phase) was retrieved, de-crosslinked and taken for analysis. The INPUT sample was first de-crosslinked and only then subjected to phenol/chloroform extraction. Equal amounts of purified DNA of both INPUT and FAIRE samples were used as templates for qPCR (INPUT is set as reference). qPCR was performed with 18 sets of primers pairs generating PCR fragments covering with partial overlaps the entire length of the IRF-8 3^rd^ intron (see S1 Table). Fold of Enrichment (FE) levels of nucleosome depleted DNA, between the FAIRE and INPUT samples were calculated for designated locations (PCR segment) across the IRF-8 3^rd^ intron and an averaged FE was calculated for each cell line separately.

### Fast Chromatin Immuno-Precipitation (Fast-ChIP)

The protocol was adopted from Nelson *et al*. [16]. Briefly, cells were crosslinked with formaldehyde and lysed. Genomic DNA was sheared using Covaris E220 (Covaris, Inc.) and precipitated using monoclonal antibodies recognizing specific histone PTMs. The following antibodies were used: αH3K27ac (ab4729, Abcam), αH3K27me3 (17–622, Upstate), anti-normal mouse IgG (ChIP-Ab and kit, Upstate) and anti-normal rabbit IgG (ChIP-Ab and kit, Upstate). Following IP the sample was de-crosslinked and DNA purified. The enriched de-crosslinked DNA samples were subjected to qPCR with 18 primers pairs as described under FAIRE. This resulted in the Fold of Enrichment of a specific antibody relative to mock IP (mouse or rabbit IgG) over a designated location (PCR segment) across the IRF-8 3^rd^ intron and an averaged FE was calculated for each cell line separately.

### Real Time PCR

The primers used for real-time PCR were designed using PrimerExpress^™^ software (Applied Biosystems, Foster City, California, USA) (see S1 Table). Primers for Tie2 and CD34 were described previously [17, 18]. One μg of total RNA was reverse transcribed to cDNA using High Capacity cDNA Reverse Transcriptase kit (Ambion, Austin, Texas, USA) according to the manufacturer’s protocol. cDNA was amplified with two primers for each gene using Power SYBR Green PCR Master Mix (Applied Biosystems) and Applied Biosystems 7300 real-time PCR System according to the manufacturer’s instructions. The amplification reaction condition was 95°C for 15 min followed by 40 cycles of 95°C for 10 s, 60°C for 20 s and 72°C for 15 s. The estimated amount of transcripts was normalized to GAPDH mRNA expression. The data is presented as the relative expression of the gene of interest compared with GAPDH.

### Luciferase reporter assay

Plasmid reporter constructs were generated by PCR amplifying the IRF-8 3^rd^ intron and the GAPDH 2^nd^ intron (1720bp) with primers flanked by MluI sites (see S1 Table) and sub-cloned to pGL3 Luciferase vector (pGL3-Luc) driven by the Nramp1 promoter (-1555, detailed in [19]). The MluI site is upstream the Nramp1 promoter generating pGL3-Luc-INT3 and pGL3-Luc-GAPDHint2. These plasmids were transfected to NIH3T3 cells and reporter gene assays were performed exactly as previously described [19].

Retroviral reporter constructs were generated by PCR amplifying the pGL3 reporter cassettes described above and sub-cloning into the pMSCV retroviral vector, generating pMSCV-Luc, pMSCV-IRF8int3 and pMSCV-GAPDHint2, respectively. NIH3T3 cells were infected and reporter gene assay were performed 72 hrs later (to ensure chromosomal integration) as described above. Luciferase light unit reads were normalized to genomic retroviral copy number, as determined using qPCR with primers for Luciferase and GAPDH as reference.

### AdOx treatment

NIH3T3 cells harboring BAC IRF-8.1 construct were plated 24 hrs prior to AdOx (A7154, Sigma-Aldrich) treatment. Cells were either untreated or treated with 25 μM AdOx. 72 hrs post-treatment EGFP and IRF-8 expression were analyzed using flow cytometry as described hereafter.

### Differentiation of miPSCs to cardiomyocytes

Undifferentiated murine induced Pluripotent Stem Cells (miPSC) cells were cultured on MEF feeder layer to 50% confluence and transduced with either pMSCV-IRF8int3 or pMSCV-GAPDHint2 retroviral vectors for 24 hrs (as described above). 48 hrs later, transduced cells were selected with Puromycin (1 μg/ml) for 1 week. To induce differentiation, the standard Hanging Drops (HD) method to derive embryoid bodies (EBs) was used. Undifferentiated miPSCs transduced with either pMSCV-IRF8int3 or pMSCV-GAPDHint2 were dissociated with 0.25% trypsin EDTA (Biological Industries) and suspended in differentiation medium composed of Iscove’s modified Dulbecco’s medium (Biological Industries), 20% fetal calf serum, 1mM L-glutamine, 0.1mM Mercaptoethanol, 1%-nonessential amino acid (Invitrogen) and 1% penicillin–streptomycin (Biological Industries). Three days cultured EBs were transferred to 6 cm Petri dishes (bacteriological grade) and four days later EBs were plated on 0.1% gelatin-coated culture dishes. The appearance of spontaneous beating colonies, indicative of differentiated cardiomyocytes, was monitored under microscope. At least 50% of the differentiated cells were cardiomyocytes and the remaining were Fibroblasts. This differentiated mixed cell population did not express IRF-8 and used for further analysis such as Luciferase assay, mRNA expression level, and ChIP analysis, as described hereafter.

### Flow cytometry

Flow cytometry analysis was performed using BD LSR-II flow cytometer (BD Bioscience) and data was analyzed using Flowing Software 2 (Cell Imaging Core, Turku Centre for Biotechnology). For GMP characterization 1 μg of biotin anti-mouse CD34 (clone RAM34, eBiosience) and 0.015 μg of PE streptavidin (BioLegend) were used.

For IRF-8 staining cells were fixed in 4% paraformaldehyde, permeabilized with 0.5% saponin, blocked with 10% normal donkey serum (D9663, Sigma-Aldrich) and stained with goat anti-IRF-8 (C-19, Santa Cruz Biotechnology) (1:100 dilution) and anti-goat IgG (CFL405, Santa Cruz Biotechnology) (1:100 dilution) or with anti-goat IgG alone as control. Unstained WT NIH3T3 cells were used as negative control for EGFP.

### Statistical methods

All Experiments were performed in n≥3 replicates and values are presented as means ± AVEDEV. Data were compared by unpaired two-tailed Student's t-test; p values <0.05 or <0.01 were considered to be statistically significant, as indicated in the appropriate figure. When applicable we employed False Discovery Rate (FDR) correction for multiple hypotheses testing, using the Benjamini-Hochberg method [20]. Asterisk indicates P-values that are significant after correction with α = 0.05 or α = 0.01.

## Results

### The third intron of IRF-8 harbors a lineage restricting regulatory element

In an attempt to identify the molecular mechanisms leading to IRF-8 repression in restrictive cells we employed BAC transgenesis [9, 21]. BACs harbor all the regulatory regions as well as the *cis*-elements and regain original chromatin architecture in a given cell or tissue allowing for authentic expression of a gene of interest regardless of their integration point [9, 22]. To generate IRF-8 BAC reporter constructs, we used the BAC genomic clone RP24-7H10, which is 219,907bp long harboring the entire murine IRF-8 gene (20,319bp) and additional upstream sequence of 118,857bp and downstream sequence of 80,731bp. Two BAC-IRF-8 reporter constructs were initially generated by inserting a reporter cassette containing a fluorescent reporter gene and an independently transcribed selectable marker (see schematic illustrations in Fig 1). In the first construct, BAC-IRF-8.1, the reporter gene cassette was inserted at the translation start site of IRF-8, and in the second construct, BAC-IRF-8.2, the whole IRF-8 coding region from translation start site to the stop codon was replaced with the reporter cassette. These two BAC-IRF-8 reporter constructs were transfected to an IRF-8 hematopoietic expression permissive cell line, the macrophage cell line RAW264.7, as well as to the non-hematopoietic expression restrictive fibroblast cell line NIH3T3. IRF-8 expression level in permissive cells is relatively low and as indicated above, can be further augmented upon exposure to IFN- γ. Accordingly, cells harboring the BAC-IRF-8 constructs were treated with IFN- γ to enhance the fluorescence intensity of IRF-8 reporter gene. In general, at least 10 different clones, harboring the various BAC-IRF-8 constructs, were isolated and the copy number was determined by quantitative real time PCR. Up to 20 copies of integrated BAC constructs were identified in the various clones and linear differences in fluorescence output were observed in direct correlation to the copy number of integrated BACs. In each set of experiments described in this section, 3 different clones harboring 1–2 copies of integrated BACs were further studied and the fluorescence intensity of the reporter gene before and following exposure to IFN-γ was visualized under fluorescent microscope and quantify as detailed under Materials and Methods. In addition, RNA was extracted from the cells and relative mRNA levels of the reporter gene and the endogenous IRF-8 gene were determined by qRT-PCR (Fig 1, ii section of each panel). Statistical analysis of fluorescence intensity of 3 independent clones expressing the various BAC constructs are detailed in Fig 1 in the i section of each panel. Fluorescent cell images of representing clones described in Fig 1 are shown in S2 Fig. It is clear from Fig 1A that in the IRF-8 permissive RAW macrophage cell line transfected with the BAC-IRF-8.1 reporter, both the BAC driven reporter gene (fluorescence) and the endogenous IRF-8 (mRNA levels) were induced by IFN-γ (Fig 1Ai and 1Aii, respectively). Similarly, IRF-8 and the reporter gene were induced in macrophage cells transfected with the BAC-IRF-8.2 construct (Fig 1B). As expected, both the BAC-IRF-8.1 driven reporter gene and the endogenous IRF-8 were not expressed in the restrictive cell line NIH3T3 (Fig 1Ai and 1Aii). Surprisingly, the reporter gene was expressed and further induced in response to IFN-γ stimulation in NIH3T3 cells transfected with the BAC-IRF-8.2 construct (Fig 1Bi and 1Bii, respectively). In contrast, the endogenous IRF-8 was not expressed in these cells (Fig 1Bii, compare gray and black columns). These results indicated that the BAC-IRF-8.1 reporter construct is authentically reporting on IRF-8 lineage restrictive expression in response to IFN-γ stimulation; fluorescent in permissive cells and not detectable in restrictive cells (see also S2 Fig). Furthermore, we could show that the lineage-specific IRF-8 expression was impaired using BAC-IRF-8.2 where the region encoding the 2^nd^ to the 9^th^ exon including intervening introns was deleted. These results suggest that the coding segment of the IRF-8 locus harbors a cell-type specific regulatory element(s) that is essential for restricting its expression in NIH3T3 cells. The open reading frame of IRF-8 is highly conserved in mammals while the intronic sequences are quite different. However, comparative sequence analysis revealed that the 1^st^, 2^nd^ and the 3^rd^ introns harbor evolutionary Conserved Nucleotide Sequences (CNSs, Fig 2). Therefore, we reasoned that the cell-type specific expression-restricting element might be confined to these CNSs. Since the non-coding 1^st^ exon and subsequent intron were not deleted in both BAC-IRF-8 reporter constructs described above, we constructed two additional BAC-IRF-8 reporter constructs in which the 2^nd^ or the 3^rd^ introns were deleted, BAC-IRF-8.3 (Fig 1C) and BAC-IRF-8.4 (Fig 1D), respectively. Deletion of the 2^nd^ intron did not affect the cell type specific repression of the reporter gene; i.e. expressed in response to IFN-γ in permissive RAW cells and repressed in restrictive NIH3T3 cells (Fig 1Ci and 1Cii). However, deletion of the 3^rd^ intron in the BAC-IRF-8.4 construct (Fig 1D) revealed that the reporter gene was expressed and further induced by IFN-γ in both RAW and NIH3T3 cells (Fig 1Di and 1Dii, florescence and mRNA levels, respectively). Collectively, these results with BAC-IRF-8.4 construct are similar with the expression pattern of the reporter gene in the BAC-IRF-8.2 construct in which the whole coding region was deleted. Taken together, these results suggest that the cell-type repression element of IRF-8 is confined to the 3^rd^ intron. Furthermore, it suggests that the cell-type specific expression of IRF-8 is governed by an active repression mechanism in restrictive cells.

### Fine deletions performed within the IRF-8 3^rd^ intron alleviated expression in restrictive cells

**Fig 3:**
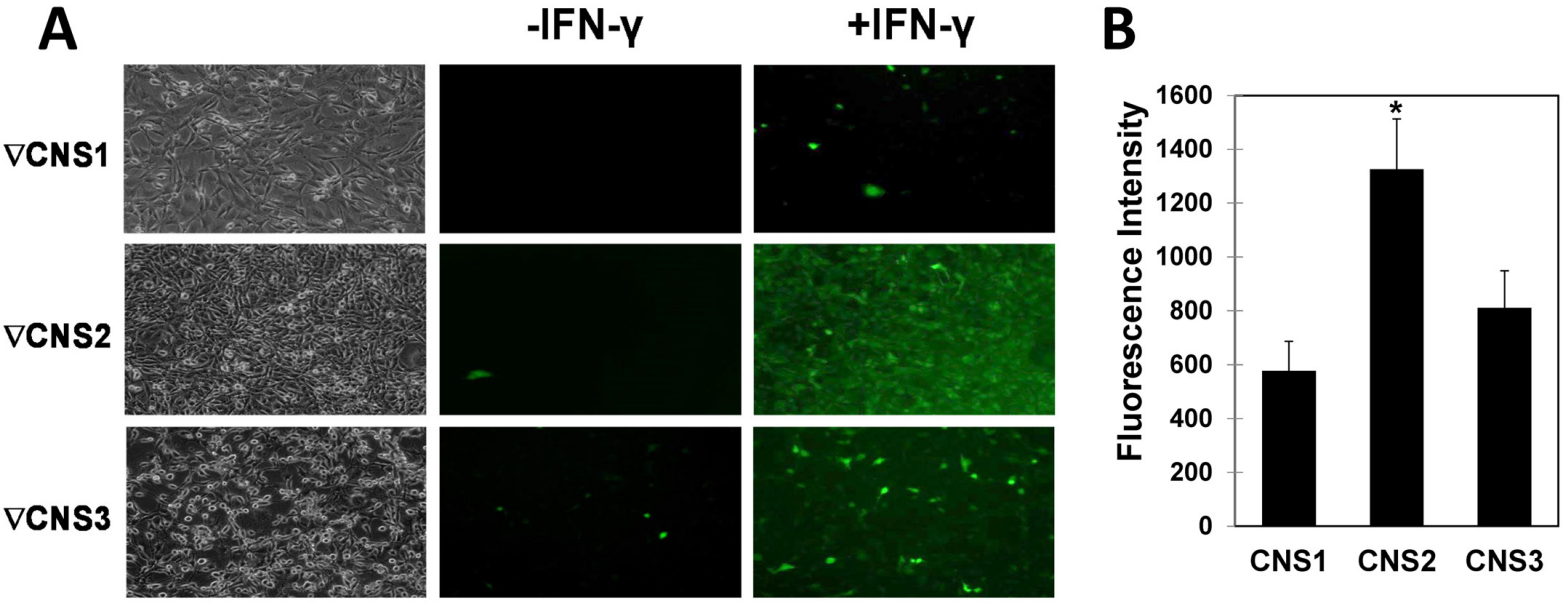
Fine deletions of the CNSs within IRF-8 3^rd^ intron. (**A**) BAC-IRF-8.1 constructs harboring deletions in conserved regions were transfected to restricted NIH3T3 cells as described under Fig 1 and stable clones were isolated. Representative clones were plated and left either untreated or treated with IFN-γ (100 U/ml) for 16 hrs and EGFP fluorescence was observed by microscopy. Deletion of CNS1 (position 1–284, upper panel), deletion of CNS2 (position (680–860, middle panel) and deletion of CNS3 (Position1230-1730, lower panel) is indicated. (**B**) GFP fluorescence intensity was determined in NIH3T3 clones transfected with various BAC IRF-8 constructs harboring CNS deletions as described under Fig 1. Values are mean ± AVEDEV (n = 3). Asterisk indicates P-values that are significant after FDR correction for multiple hypotheses testing with α = 0.05.

In an attempt to identify the exact regulatory element(s) within the IRF-8 3^rd^ intron that leads to gene repression, fine deletions of the conserved regions were performed. As seen in Fig 2, bioinformatics analysis of the 1730bp long 3^rd^ intron, using ECR Browser [23], revealed three CNSs: CNS1, 2 and 3. In each CNS there are one or two dense clusters harboring putative Transcription Factors (TF) binding motifs (MatInspector, Genomatix Software Suite [24]). To create constructs with CNSs deletion VCre-mediated recombination was employed (see Materials and Methods for details). The BAC-IRF-8.1 construct, harboring the EGFP cassette inserted to the first methionine of the IRF-8 gene, was used to evaluate the effect of such fine deletions. Three different deletions encompassing the three CNSs were performed; deletions of CNS1 (position 1–284), CNS2 (position 680–860) and CNS3 (position 1230–1730). Following VCre mediated removal of the antibiotic cassette, the new BAC constructs were transfected to restrictive cells and stable clones were further characterized; 7 clones from deletion of CNS1, 9 clones from deletion of CNS2 and 7 clones from deletion of CNS3. The EGFP fluorescence in each clone was evaluated by microscopy before and following induction with IFN-γ. Reporter gene fluorescence of representative clone of each CNS deletion is shown in Fig 3A and compiled data for 3 representing clones for each deletion is presented in Fig 3B. As shown in Fig 3, deletion of CNS2 (Fig 3A, mid panel and Fig 3B) exhibited a significantly higher level of EGFP fluorescence in comparison to the other two deletions. Further, the deletion of CNS3 (Fig 3A, lower panel and Fig 3B) exhibited mid-level EGFP intensity in comparison to the deletion of CNS1, which exhibited the lowest fluorescence level (Fig 3A, top panel and Fig 3B). Together, deletion of each of the three CNSs within this intron revealed a gradual alleviation of repression of the IRF-8 reporter gene; the strongest alleviation was noted with CNS2 deletion while the weakest with CNS1.

### Nucleosome occupancy and repressive chromatin architecture is enriched over the IRF-8 3^rd^ intron in expression restrictive cells

**Fig 4:**
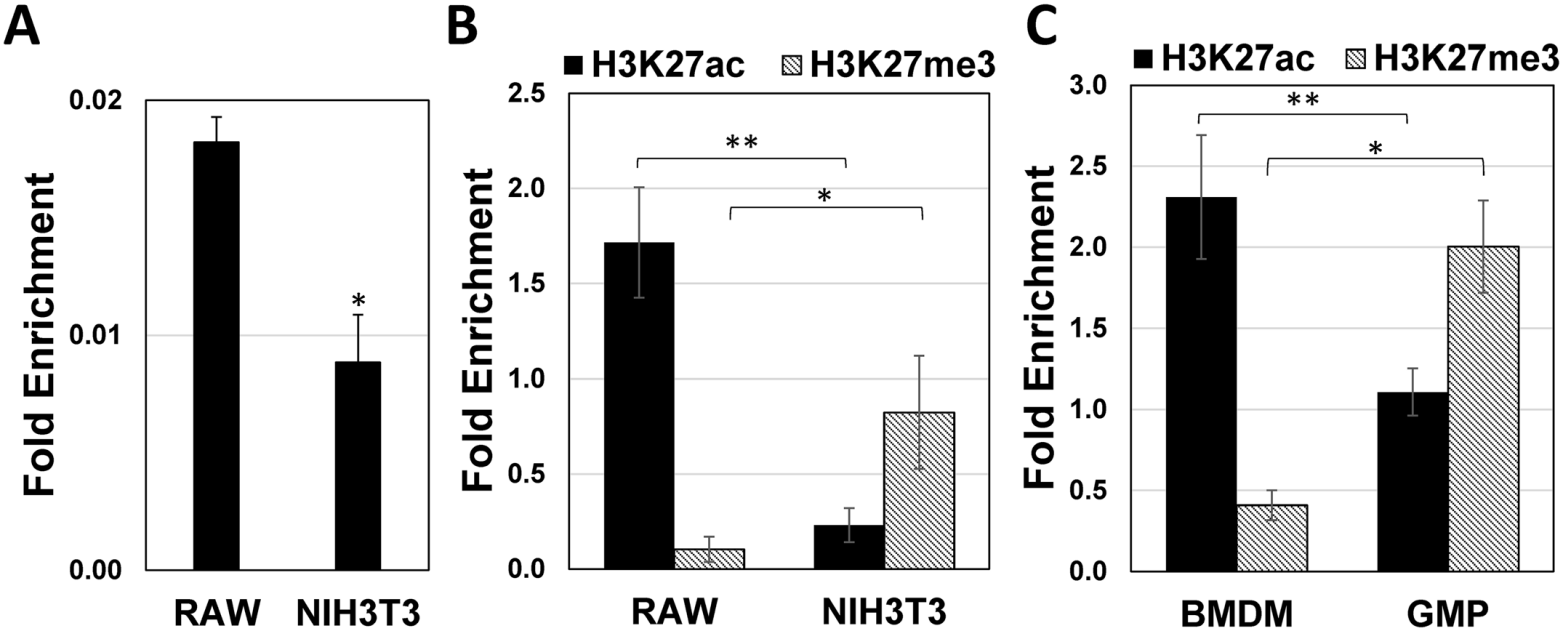
Differential nucleosome occupancy and histone PTM profile across the IRF-8 3^rd^ intron between RAW and NIH3T3 cells. (**A**) Differential nucleosome occupancy across the IRF-8 3^rd^ intron between RAW and NIH3T3 cells. RAW, NIH3T3 (**B**) as well as bone marrow derived GMP and BMDM cells (**C**) were subjected to ChIP-qPCR using histone modification monoclonal antibodies directed against H3K27ac (black bars) and H3K27me3 (gray bars). Values are means ± AVEDEV (n = 3) and calculated as fold enrichment compared with mock IP (normal IgG) and normalized for DNA quantity against fold enrichment at the GAPDH gene. Asterisks represent statistical significance (Students t-test, * *p*<0.05, ** *p*<0.01).

The BAC reporter results clearly indicated that the repression of IRF-8 expression in restrictive cells is mediated by its third intron. In order to characterize the molecular mechanism governing this lineage-specific restriction, we explored the involvement of chromatin architecture. Initially, differences in nucleosome occupancy over IRF-8 3^rd^ intron between RAW and NIH3T3 cell lines were analyzed using formaldehyde-assisted isolation of regulatory elements (FAIRE) technique [15]. This is an alternative approach to DNaseI hypersensitivity assay aiming at identifying DNA regulatory elements that are evicted of nucleosomes (for details see Materials and Methods). The data in Fig 4A is presented as averaged Fold Enrichment of nucleosome-depleted DNA in the RAW and NIH3T3 cell lines across the entire IRF-8 3^rd^ intron. The results clearly demonstrate that the 3^rd^ intron is more depleted of nucleosomes in the hematopoietic IRF-8 permissive cell line, RAW, in comparison to the non-hematopoietic cell line, NIH3T3 (Fig 4A). This indicates that open chromatin structure of the IRF-8 3^rd^ intron is associated with IRF-8 permissiveness.

We next tested whether the difference in chromatin architecture between IRF-8 permissive and restrictive cells revealed by FAIRE is also supported by differential histone PTM profiles. For that purpose, we compared histone 3 lysine 27 tri-methylation (H3K27me3), a modification correlated with dense chromatin architecture [25–27], to histone 3 lysine 27 acetylation (H3K27ac) that is correlated with open chromatin architecture [28]. The data in Fig 4B clearly shows that H3K27me3 modification is highly enriched in IRF-8 3^rd^ intron in NIH3T3 cells in comparison to RAW cells while the opposite was observed for the H3K27ac modification; enriched in RAW cells. Together, these results support the FAIRE data and point to a dense chromatin architecture in the IRF-8 3^rd^ intron in restrictive cells.

To verify these results in a primary cell system, we also analyzed the abundance of these chromatin modifications in murine primary hematopoietic restricting cells such as GMP cells in comparison to permissive BMDM cells. BM cells were harvested and grown under conditions supporting the differentiation of these two cell types (detailed under Materials and Methods). Seven days later, ChIP analysis across IRF-8 3^rd^ intron in these cell types was performed as detailed in Fig 4C. The two tested histone PTMs exhibited differential enrichment patterns between GMP (restrictive) and BMDM (permissive) cells. Histone PTM H3K27me3, associated with condensed chromatin architecture, exhibited higher enrichment level in GMPs (Fig 4C). Conversely, histone PTM H3K27ac, associated with open chromatin architecture, exhibited higher enrichment levels in BMDM cells (Fig 4C). Taken together, this epigenetic “signature” exhibited in cell lines and primary bone marrow derived cells indicates that specific chromatin remodeling of the IRF-8 3^rd^ intron is a hallmark of myelopoiesis in general and differentiation of GMP cells to the monocyte\macrophage lineage in particular.

### Inhibition of H3K27me3 PTM leads to partial alleviation of IRF-8 suppression in restrictive cells

**Fig 5:**
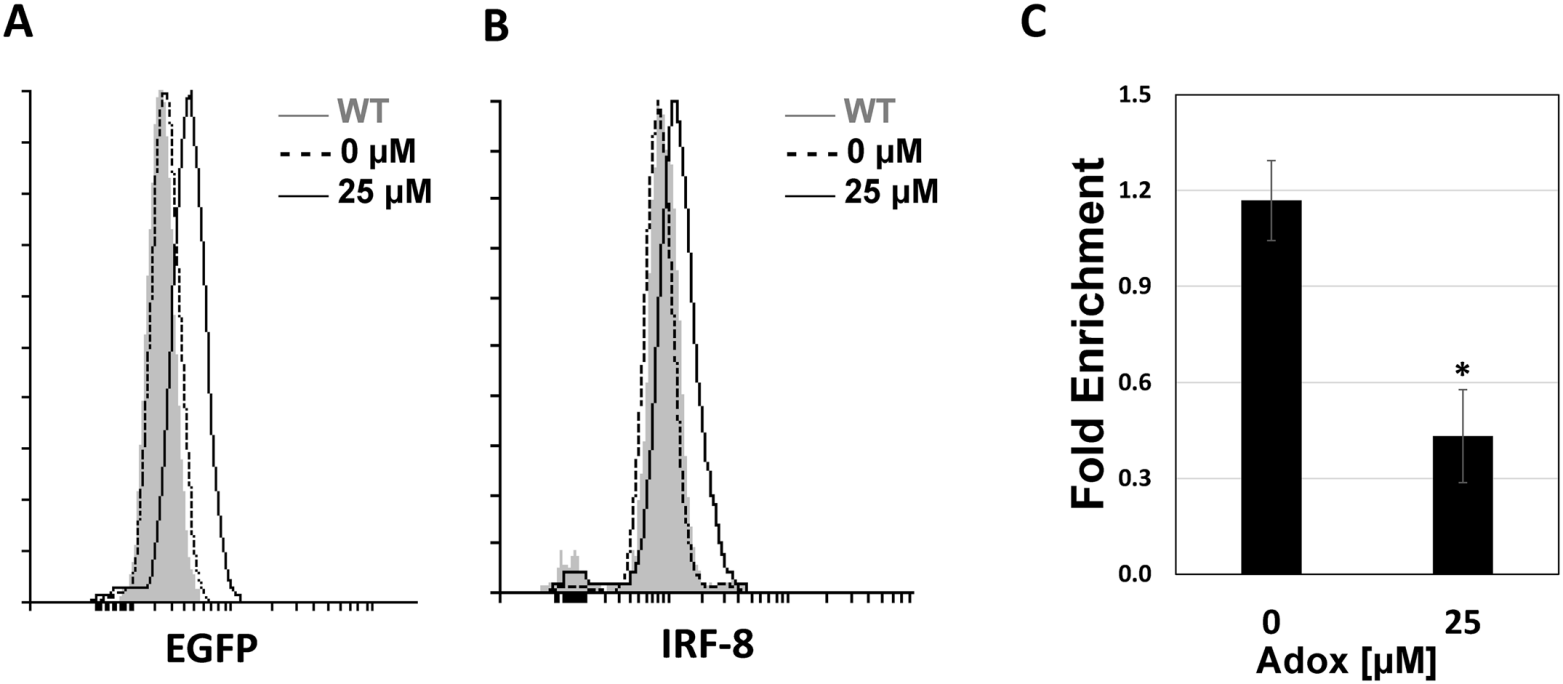
AdOx treatment alleviated both IRF-8 reporter gene and the endogenous IRF-8 in NIH3T3 restricting cells. NIH3T3 cells harboring BAC-IRF8.1-EGFP construct were either left untreated or treated with 25μM AdOx. EGFP (**A**) and IRF-8 (**B**) expression were measured using flow cytometry (for details see Materials and Methods). Cells exhibiting high EGFP expression were sorted and subjected to ChIP analysis (**C**) using monoclonal antibodies directed against H3K27me3 and fold enrichment was calculated as described under Fig 4. Asterisks represent statistical significance (Students t-test, * *p*<0.05).

H3K27 tri-methylation is mediated by Enhancer of Zeste Homolog 2 (EZH2), which is the catalytic subunit of Polycomb Repressive Complex 2 (PRC2) [29, 30]. To test the possibility that inhibition of this modification will affect chromatin architecture of the IRF-8 3^rd^ intron resulting in alleviated IRF-8 expression we used Adenosine dialdehyde (AdOx), a general inhibitor of S-adenosylmethionine (AdoMet)-dependent methyltransferases [31]. Addition of AdOx to NIH3T3 cells harboring the BAC-IRF-8.1 reporter construct alleviated the restriction on the reporter gene expression as well as on the endogenous IRF-8 expression (Fig 5A and 5B, respectively). A significant shift in the fluorescence peaks corresponding to the EGFP (Fig 5A) and the anti-IRF8 staining (Fig 5B) can be observed in AdOx treated NIH3T3 cells.

To demonstrate that the non-specific methyltransferase inhibitory activity of AdOx also affected the level of H3K27me3 PTM, cells were treated with AdOx as described above and cells exhibiting significant shift in the intensity of EGFP were sorted and subjected to ChIP analysis. Significant depletion of H3K27me3 modification over the IRF-8 third intron was evident (Fig 5C) underlining the effect of AdOx on H3K27me3 PTM.

Together, these results indicate that the suppressive histone PTM, H3K27me3, is part of the regulatory mechanism leading to IRF-8 active repression. Additionally, the concurrent change in both the endogenous IRF-8 and its EGFP reporter gene underlies the authentic report of BAC-IRF-8.1 construct and the validity of BAC transgenesis as a reliable reporter system.

### IRF-8 3^rd^ intron serves as an initiator of gene repression in restrictive cells

**Fig 6:**
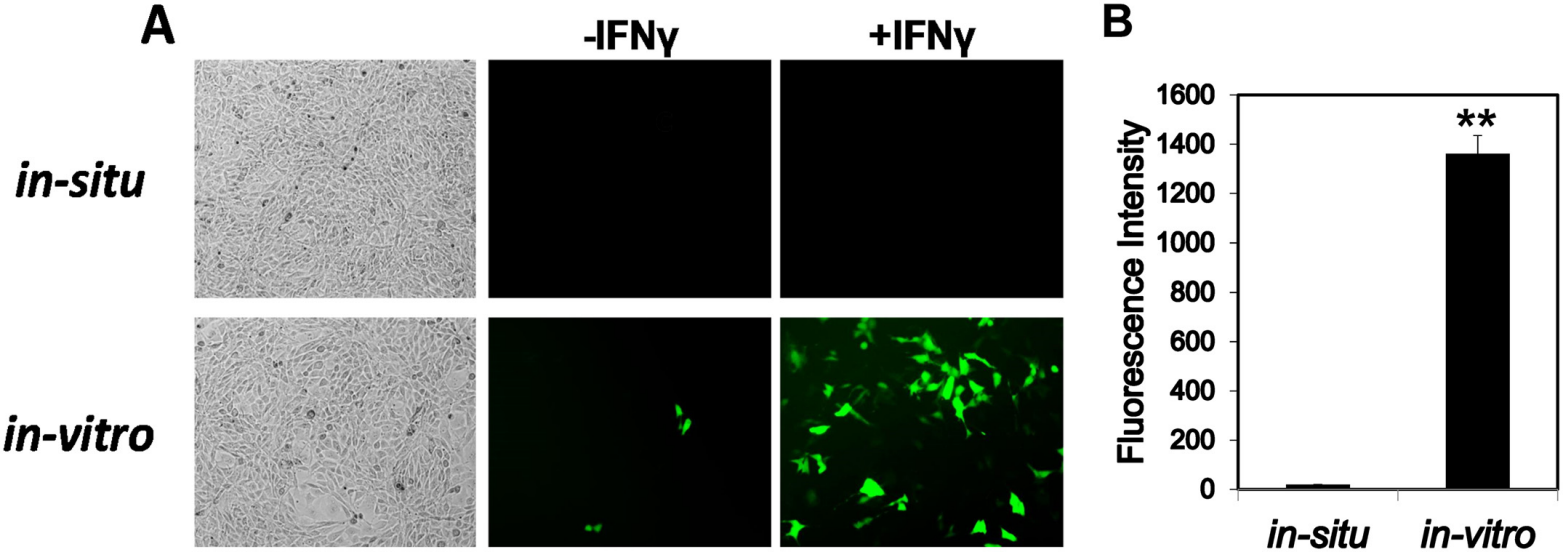
Deletion of the IRF-8 3^rd^ intron prior to transfection to restrictive cells alleviated the reporter repression. (**A**) NIH3T3 were transfected with BAC-IRF-8.1 VLoxP. To induce 3^rd^ intron deletion within the cells (*in-situ*), stable clones were transduced with a retroviral vector encoding the VCre gene. (**B**) The 3^rd^ intron in BAC-IRF-8.1 VLoxP construct was initially deleted with the corresponding VCre recombinase in *E*. *coli* (*in-vitro*). Subsequently the corresponding BAC DNA was transfected to NIH3T3 and stable clones were selected. A representative clone harboring 1–2 BAC copies is shown in each panel. Clones were induced with IFN-γ (100 U/ml) for 16 hrs and EGFP fluorescence was observed by microscopy. (**C**) EGFP fluorescence intensity was determined in NIH3T3 clones in which the IRF-8 3^rd^ intron was deleted *in-situ* or *in-vivo* (for details see Materials and Methods). Values are mean ± AVEDEV (n = 3). (Students t-test, ** p<0.01).

In order to analyze the reporter gene expression in a single cell clone before and following removal of the 3^rd^ intron, we constructed a new BAC reporter construct, BAC-IRF8.1-VLoxP. Since our BAC constructs already contain both classical Cre/LoxP as well as FLP-FRT site [13], specific recombination systems, we utilized a new site-specific recombination system, VCre/VLoxP [14]. A new BAC construct in which the IRF-8 3^rd^ intron is flanked by two VLoxP sites was created. This construct was transfected to restrictive NIH3T3 cells and five cell clones were isolated. To perform 3^rd^ intron deletion within the cells, clones were transduced with either empty retroviral vector or retroviral vector coding for the VCre gene (detailed under Materials and Methods), and EGFP fluorescence intensity was subsequently analyzed by either fluorescence microscopy and fluorescence quantitation (Fig 6A and 6B, respectively) or by determining mRNA levels of both EGFP and endogenous IRF-8 (S3 Fig, *in-situ*). Surprisingly, despite the fact that the intron was removed in clones harboring BAC-IRF8.1-VLoxP construct, no reporter expression was detected by flow cytometry (data not shown) or by fluorescence microscopy, even following treatment with IFN-γ (representing clone in Fig 6A and compiled analysis of 3 independent clones in Fig 6B). We hypothesized that since the BAC construct was initially transfected with an intact 3^rd^ intron the IRF-8 locus within this BAC construct already gained condensed chromatin architecture in the restrictive cells, followed by yet undefined "epigenetic memory". Therefore, the subsequent removal of the 3^rd^ intron within the cells had no effect on the expression and had no effect on the architecture state of the repressed chromatin. Even ectopic expression of PU.1, essential for IRF-8 expression [32], had no effect (data not shown). To prove this hypothesis, we deleted the 3^rd^ intron using VCre in bacteria and subsequently transfected the resulting BAC into NIH3T3 cells. Stable clones were isolated and EGFP fluorescence intensity was evaluated as described above before and following treatment with IFN-γ (representing clone in Fig 6A and complied data for 3 independent clones in Fig 6B). It is clear that removal of the 3^rd^ intron prior to transfection led to the expression of the reporter gene in the restrictive cells. This was also evident at the mRNA level of both the reporter gene and the endogenous IRF-8 (S3 Fig, *in-vitro*). These results recapitulate our findings summarized in Fig 1D in which the 3^rd^ intron had been swapped with antibiotic expression cassette prior to the transfection to cells. The difference between these two experiments is the fact that the 3^rd^ intron was "surgically" removed and not swapped with a selectable marker cassette. The fact that removal of the 3^rd^ intron within restricting cells was not sufficient to alleviate repression indicates that this sequence is needed to initiate IRF-8 repression but is dispensable for sustained repression.

**Fig 7:**
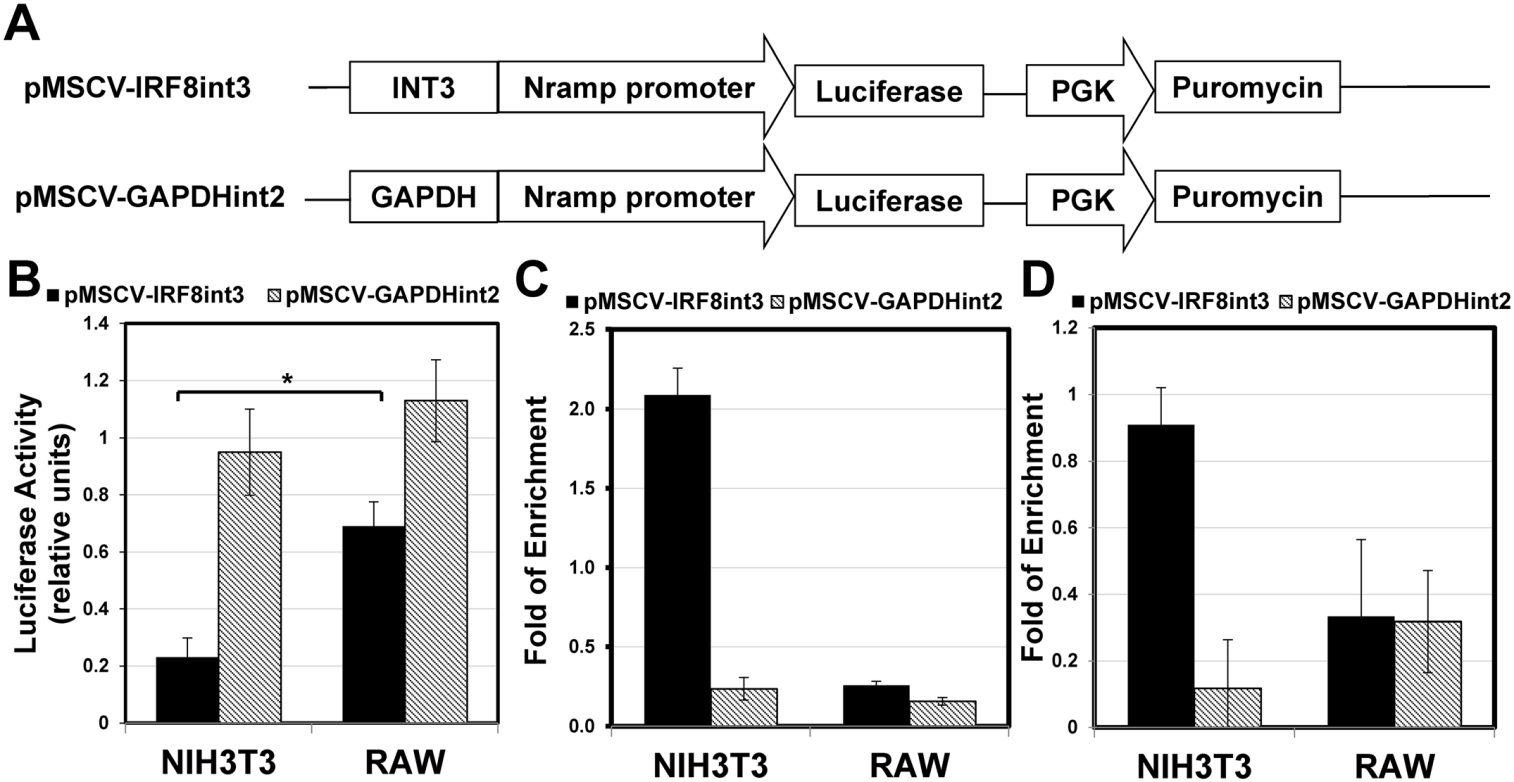
IRF-8 3^rd^ intron is sufficient to repress reporter gene expression in restrictive cells. (**A**) NIH3T3 and RAW cells were transduced with constructs harboring either IRF-8 3^rd^ intron or GAPDH 2^nd^ intron upstream to the reporter gene as schematically illustrated (pMSCV-IRF8int3 and pMSCV-GAPDHint2, respectively). (**B**) Luciferase expression levels were determined 72 hrs post transduction. pMSCV-Luc Luciferase expression was determined as 1. Values are in relative light unites, means ± AVEDEV (n≥3). ChIP-qPCR analysis of H3K27me3 modification at the Luciferase reporter gene (**C**) and Puromycin gene (**D**) in NIH3T3 or RAW cells transduced with either pMSCV-IRF8int3 or pMSCV-GAPDHint2 constructs was performed using different three primer pairs from luciferase and Puromycin genes (Luciferase 1, 2,3, and Puromycin 1,2,3, S1 Table, respectively). Values are means ± AVEDEV (n = 3) and calculated as described under Fig 4. Asterisk indicates P-values that are significant after FDR correction for multiple hypotheses testing with α = 0.05.

To further establish the role of the IRF-8 3^rd^ intron as initiator of repression of the IRF-8 locus, we cloned it upstream of a Luciferase reporter gene in the reporter plasmid pGL-3. As a control, we also cloned the 1720bp of the 2^nd^ intron of GAPDH. These three reporter plasmids (pGL3, pGL3-INT3, pGL3-GAPDHint2, detailed under Materials and Methods) were transiently transfected in NIH3T3 cells. However, no significant differences in Luciferase activities between these three reporter constructs were noted (data not shown). We reasoned that in these transient transfection assays, the plasmids do not assemble proper chromatin conformation. Therefore, the IRF-8 3^rd^ intron and the GAPDH 2^nd^ intron were sub-cloned into a retroviral vector, pMSCV, upstream to the reporter gene as illustrated in Fig 7A and detailed under Materials and Methods. To ensure chromosomal integration, NIH3T3 cells were harvested 72 hrs following infection. Luciferase assay was performed and the data was calibrated against cell number (protein level) and retroviral transduction efficiency (for details see Materials and Methods). The Luciferase activity of the cells transduced with the retroviral vector harboring the reporter gene and the 3^rd^ intron upstream to its coding sequence, pMSCV-IRF8int3, exhibited significant decrease in the reporter gene activity (~5 fold) in comparison with the control vector pMSCV-GAPDHint2 (Fig 7B). Conversely, no such significant inhibition of the reporter gene was noted with the same retroviral vectors in the IRF-8 permissive macrophage cell line RAW (Fig 7B). Moreover, ChIP analysis of H3K27me3 PTM over the transduced Luciferase gene indicated that the IRF-8 3^rd^ intron elicited a repressed chromatin state. This was evident by the significant enrichment of H3K27me3 PTM enrichment only in IRF-8 expression restrictive NIH3T3 cells and not in transduced IRF-8 expression permissive RAW cells (Fig 7C). Additionally, no change in H3K27me3 PTM was noted with reporter construct harboring the 2^nd^ intron of GAPDH between the two cell types (Fig 7C). Taken together, our data suggest that this intronic element acts as initiator of repressed chromatin state on naked DNA only in IRF-8 expression restrictive cells. Interestingly, when antibiotic selection was applied on NIH3T3 cells to select for infected cells, the efficiency of transduction of only pMSCV-IRF8int3 was sharply reduced while that of pMSCV-Luc and pMSCV-GAPDHint2 was similar (data not shown). Consequently, the above-mentioned assays, Luciferase and ChIP were performed 72 hrs after transduction (Fig 7B and 7C, respectively) without selection pressure to exclude this bias. Furthermore, ChIP analysis clearly indicated the IRF-8 3^rd^ intron elicited repressed chromatin state over the distal Puromycin resistance gene driven by a different promoter as was evident by the enrichment of H3K27me3 only in NIH3T3 cells (Fig 7D). Taken together, these results point to the ability of the 3^rd^ intron to induce effective local gene silencing only in IRF-8 expression restrictive cells when the integrating viral DNA gained chromosomal conformation. Furthermore, despite a certain bias of retroviral integration, multitude sites along the transduced cells genome are targeted. Therefore, these results point to the general ability of the 3^rd^ intron to elicit repressed chromatin independent of integration site.

### The IRF-8 3^rd^ acts as initiator of repressed chromatin only in differentiated iPSCs cells

**Fig 8:**
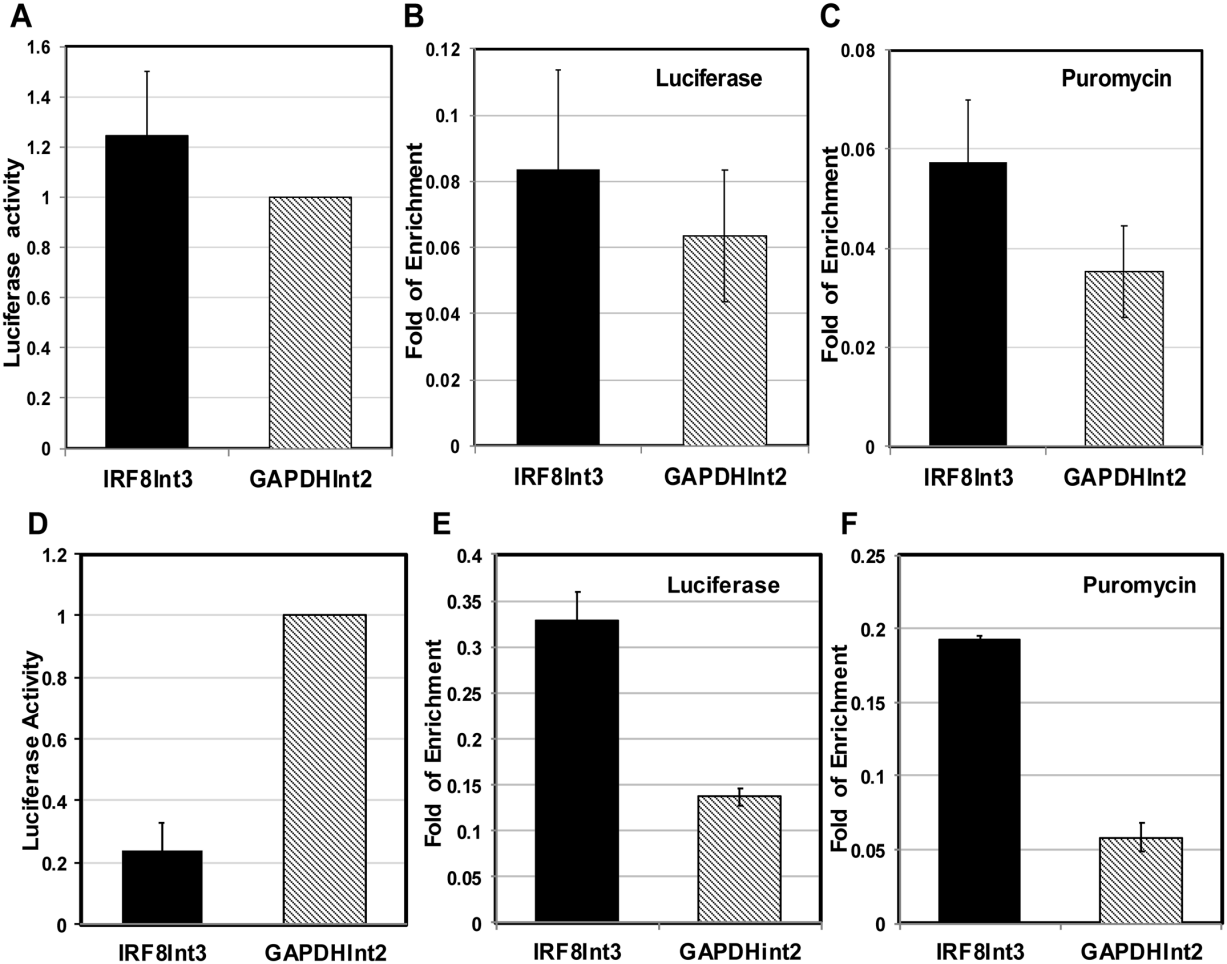
IRF-8 3^rd^ intron has no effect on expression level and chromatin state of the transduced Luciferase and Puromycin genes in naïve iPSCs. iPSCs were transduced with either pMSCV-IRF8int3 or pMSCV-GAPDHint2) reporter constructs. Subsequently, cells were further differentiated to cardiomyocytes (as detailed in Materials and Methods). Luciferase levels were measured and relative Luciferase activity was calculated for the undifferentiated and differentiated iPSCs ((**A**) and (**D**), respectively). ChIP-qPCR analysis of H3K27me3 modification was performed on undifferentiated and differentiated iPSCs at the Luciferase reporter gene ((**B**) and **(E**), respectively) and the Puromycin gene ((**C**) and (**F**), respectively). Values are means ± AVEDEV (n = 3) and fold enrichment was calculated as described under Fig 4.

To test the ability of IRF-8 3^rd^ intron to act as an initiator of repressed chromatin, we turned to iPSCs that maintain naïve or poised chromatin architecture. These cells are subjected to massive chromatin rearrangement upon differentiation [10]. We expected that the IRF-8 3^rd^ intron upstream to a reporter gene in a retroviral vector system described above (see schematic illustration in Fig 7A) will not exert any repressive state on the adjacent reporter gene or on the distal Puromycin resistance gene. However, upon differentiation of the same transduced iPSCs into cardiomyocytes (as detailed under Materials and Methods) the 3^rd^ intron will elicit repression of the reporter gene as well as the distal Puromycin resistance gene. Indeed, it is clear from Fig 8A that the relative Luciferase expression is similar between iPSCs cells that were transduced with the retroviral reporter construct harboring either IRF-8 3^rd^ intron or GAPDH 2^nd^ intron. Similar expression was also observed at the mRNA level of both the Luciferase gene and the Puromycin resistance gene between the IRF-8 3^rd^ intron and GAPDH 2^nd^ intron transduced cells (S4A Fig, IRF8int3 and GAPDHint2, respectively). Finally, ChIP analysis of H3K27me3 PTM level over the Luciferase gene and the Puromycin gene were, as expected from naïve chromatin, similar and very low as fold of enrichment scale is lower than 0.1 (Fig 8B and 8C, respectively). However, when the same transduced cells were triggered to differentiate toward cardiomyocytes, a significant difference in the expression level of the reporter gene between cells transduced with IRF-8 3^rd^ intron and GAPDH 2^nd^ intron was evident. A fivefold reduction in luciferase activity was noted in cells transduced with the 3^rd^ intron (Fig 8D, IRF8int3 and GAPDHint2, respectively). Accordingly, 3–5 fold reduction in the mRNA levels corresponding to both Luciferase and Puromycin resistance genes were noted in these 3^rd^ intron transduced cells (S4B Fig, IRF8int3). Concomitantly, the IRF-8 3^rd^ intron initiated a repressed chromatin state as was evident by the elevate H3K27me3 PTM level for both the Luciferase gene and the more distal Puromycin resistance gene in IRF-8 3^rd^ intron transduced cells in comparison the GAPDH 2^nd^ intron transduced cells (Fig 8E and 8F, respectively). Although retroviruses integrate mainly into open-chromatin-structures [33], only the presence of the IRF-8 3^rd^ intron elicited repressed chromatin state of the integrated retroviral-vector irrespective of integration site that is different in each transfected cell. It is important to mention that like NIH3T3 cells, this differentiated cell population is also restrictive for IRF-8 expression (S5 Fig). Together, our data clearly demonstrate the ability of the IRF-8 3^rd^ intron to act as an initiator of repressed chromatin when naïve chromatin undergoes massive architectural changes that accompany differentiation. This repression initiation capacity is independent of the integration site.

## Discussion

IRF-8 is a member of the IRF family, which is expressed in a lineage-restricted manner and plays a key role in lineage commitment, cell type development, and functionality of mature macrophages, dendritic cells and B-cells [34–37]. Mice with IRF-8 null mutation are defective in the ability of myeloid progenitor cells to mature towards the macrophage lineage and eventually develop Chronic Myelogenous Leukemia (CML)-like syndrome. Taken together, IRF-8 acts both as an orchestrating factor of myeloid cell differentiation and as a myeloleukemia suppressor gene. Its expression is strictly limited to the aforementioned cell types. This study aimed at mechanistically elucidating how IRF-8 expression is excluded from expression restricting cells.

Using BAC technology, we clearly show that intragenic DNA sequences govern repression of IRF-8 expression in restricting cells. This points to a unique regulatory element in a non-coding intronic segment that has global effect on IRF-8 expression, possibly by affecting the promoter and other intergenic regulatory elements. We show that a defined element is necessary and sufficient to silence homologous and heterologous gene expression in restricting cells (Figs 1 and 7). Interestingly, this inhibitory element is confined only to the 3^rd^ intron of IRF-8. We have found three CNS in the IRF-8 3^rd^ intron and deletion of each CNS revealed a gradual alleviation of repression of the IRF-8 reporter gene; the strongest alleviation was noted with CNS2 deletion while the weakest was detected with a deletion of CNS1 (Fig 3). This indicates a collaborative silencing effect of all three CNS. Since a repressed chromatin state is characteristic of the IRF-8 locus in general and the 3^rd^ intron in particular in restricting cells, it could suggest that interacting factors binding to each CNS may act in concert to elicit this chromatin-repressed state. Other intragenic elements affecting gene expression were described previously (reviewed in [38]). These act as enhancers of gene expression and to our knowledge, only two repressive elements within introns are known to date [39, 40].

Our results point to the possible role of IRF-8 3^rd^ intron as a nucleation core for chromatin condensation/remodeling in expression restricting cells. This is supported by the fact that only the recombinase-mediated deletion of the IRF-8 3^rd^ intron prior to transfection led to the alleviation of IRF-8 reporter gene expression, while removal of the intron after integration into the host DNA had no effect on the repressed state of the reporter gene. The latter suggests that the establishment of an "epigenetic memory" follows the initial onset of chromatin remodeling. This "memory" is likely to depend on a defined set of histone PTMs [41] that are spread along the IRF-8 locus. In this process, the IRF-8 3^rd^ intron serves as a memory recruitment sequence and its function exerted by unknown effectors. Interestingly, once epigenetic memory is established, its presence/function is no longer needed to repress IRF-8 expression. Presumably, the 3^rd^ intron serves as a cue site for the IRF-8 locus, providing a platform for cell-type specific DNA interacting factors during cell differentiation process. In restricting cells, these factors initiate histone PTMs affecting chromatin architecture that are followed by further modifications that install the epigenetic memory. This is supported by our observation that the IRF-8 3^rd^ intron is capable of repressing a Luciferase reporter gene only when inserted to a retroviral vector that randomly integrates into the genome and assembles chromatin conformation in restrictive cells. This does not take place in permissive cells or in transient transfection assays, where the transfected plasmid DNA does not assemble proper chromatin structure [42]. Taken together, these results suggest that the IRF-8 3^rd^ intron does not act as a "classical" silencer.

Our results show that the IRF-8 3^rd^ intron is able to silence a reporter gene upon differentiation of iPSCs into cardiomyocytes validating its role as an initiator of repressed chromatin in IRF-8 expression restrictive cells. Interestingly, its repressive activity cannot be exerted in undifferentiated iPSCs indicating that these cells lack the above postulated factors that recruit the repressive machinery. As such, the fold of enrichment scale of iPSCs was 10 times lower in comparison to cardiomyocytes or NIH3T3 (Figs 8B, 8E and 7C, respectively). Furthermore, our data clearly show the ability of IRF-8 3^rd^ intron to silence a proximal heterologous gene, i.e., the Puromycin resistance gene. This gene was also repressed only by the 3^rd^ intron as was evident by ChIP assays and mRNA levels (Figs 8F and S4B). The Puromycin resistance gene is driven by an independent promoter, yet the IRF-8 3^rd^ intron, which is located 3770 bp upstream, is capable of influencing its expression by modulating the chromatin state. It is important to note that the 3^rd^ intron did not elicit a complete shutdown of both the reporter gene and the Puromycin resistance gene. The reporter gene was still detectable at very low level by FACS analysis (Fig 5A) or in few cells that appear to escape repression (Figs 3 and 6). Further the mRNA level of the antibiotics resistance gene was expressed at low level (S4B Fig) that was probably sufficient to exert resistance in **some** of the cells. Together, this suggest that position effect variegation on heterochromatin may have a role in alleviating the expression of the reporter gene and consequently also the expression of the antibiotic resistance gene [43]. Together, these results point to the role of this intronic element as an initiator of *de-novo* generation of repressed chromatin over a distance of at least several kb.

It is well established that histone PTM composition is directly linked to chromatin architecture [27, 44]. Our results reveal higher nucleosome occupancy over the 3^rd^ intron of IRF-8 in restrictive cell lines that is accompanied by H3K27me3 enrichment (Fig 4). Treatment of the restrictive cell line NIH3T3 with AdOx, a broad inhibitor of S-adenosylmethionine (AdoMet)-dependent methyltransferases [31] including H3K27me3 PTM (Fig 5), significantly alleviates this restrictive phenotype. The PRC2 complex is the main machinery for H3K27 methylation highlighting its role in mediating chromatin condensation and the subsequent silencing of IRF-8 locus in restrictive cells. This is supported by the genome-wide study by Bracken *et al*. annotating PcG targets, among which is the IRF-8 locus [45]. This H3K27me3 mediated repression mechanism is functional in restrictive cells of non-hematopoietic origin as well as in myeloid progenitor cells of hematopoietic origin that are also restrictive for IRF-8 expression. Support for our results is also derived from the ENCODE project datasets [46] showing the same H3K27me3 enrichment pattern over the IRF-8 locus in restrictive cell lines such as normal human lung fibroblasts, as opposed to no enrichment in a permissive cell line, human monocytes (S6 Fig). The fact that AdOx alleviated both the endogenous IRF-8 as well as the IRF-8 BAC reporter gene expression underlies the authenticity of the BAC IRF-8 reporter system.

While *in-vivo* removal of the IRF-8 3^rd^ intron cannot alleviate its repression, molecular events taking place during differentiation are orchestrating such cell-type specific repression. The ability of transcription factors to locally influence Polycomb activity and subsequently the chromatin state was demonstrated [47, 48]. We hypothesize that a cell type specific DNA interacting factor or a combination of factors binding to the 3^rd^ intron facilitates the recruitment of the Polycomb complex. This takes place during differentiation or upon assembly of chromatin on naked\naïve (poised) chromatin. As shown here, the 3^rd^ intron alone is sufficient to elicit repressed chromatin state. The fact that each CNS contributes differently to the establishment of repressed chromatin (Fig 3), suggests that a combinatorial assembly of DNA interacting factors accounts for the recruitment of the Polycomb complex.

In conclusion, our studies on the IRF-8 3^rd^ intron suggest a model whereby multiple CNS mediate repression of IRF-8 gene expression in restrictive cells even in the presence of activating stimuli (e.g. IFN- γ). These CNS recruit directly or indirectly the chromatin repressive epigenetic machinery. We hypothesize that this repressive effect is subsequently spread along the IRF-8 locus in an undulation motion. It is tempting to speculate that similar mechanisms repress other genes specific for macrophages, DCs, and B-cells and sequence comparisons will need to identify those elements. Future studies may make use of the CNS identified here to experimentally achieve specific transgene repression in IRF-8 restrictive cells, e.g. in transgenic animals.

## Supporting Information

S1 Fig Characterization of primary mouse bone marrow derived GMP and BMDM cells. BM cells were harvested from the tibia and femur of 6–8 weeks old C57BL/6 mice, and cultivated with medium supplemented with IL-3 or M-CSF, resulting in GMP (CD34^high^) and BMDM cells, respectively. Cell characteristics were determined by analyzing GMP associated gene markers, CD34 and Tie2 [49, 50] AKA and macrophages associated marker, M-CSF receptor [51]. (**A**) Flow cytometry analysis of cell surface marker CD34 on GMP cells. qRT-PCR was employed to determine relative gene expression levels of CD34 (**B**), Tie2 (**C**) and M-CSF receptor (**D**). Expression level in BMDM cells was determined as 1. (**E**) Cells were treated with IFN-γ (100U/ml for 16 hrs. and IRF-8 induced expression in GMP and BMDM cells was calculated. IRF-8 expression level in untreated cells was determined as 1. Results shown are mean ± AVEDEV (n = 3).
(PDF)

S2 Fig Reporter gene expression in representative clones harboring BAC-IRF-8 constructs. RAW and NIH3T3 cells were transfected with the various BAC constructs and the fluorescence activity of the reporter gene in representative RAW and NIH3T3 stable clones, harboring 1–2 copies of the BAC reporter construct, was visualized under fluorescent microscope before and following 16 hrs of exposure treatment with IFN-γ (100 U/ml). Representative clones harboring BAC-IRF-8.1(**A**), BAC-IRF-8.2(**B**), BAC-IRF-8.3 (**C**) and BAC-IRF-8.4 (**D**) are shown.
(PDF)

S3 Fig mRNA expression levels of EGFP and IRF-8. NIH3T3 were transfected with BAC-IRF-8.1 VLoxP as described under Fig 6. To induce 3^rd^ intron deletion within the cells (*in-situ*), stable clones were transduced with a retroviral vector encoding the VCre gene. For *in-vitro* deletion, the 3^rd^ intron in BAC-IRF-8.1 VLoxP construct was initially deleted with the corresponding VCre recombinase in *E*. *coli* and subsequently transfected to NIH3T3 and stable clones were selected. The mRNA levels of the reporter gene (EGFP) and the endogenous IRF-8 were determined by real-time q-PCR from three independent clones for each deletion type; *in-situ* and *in-vitro*. Values are mean ± AVEDEV (n = 3).
(PDF)

S4 Fig Luciferase reporter gene and Puromycin relative mRNA expression in undifferentiated and differentiated miPSCs. miPSCs were transduced with either pMSCV-IRF8INT3 (IRF8Int3) or pMSCV- GAPDHint2 (GAPDHint2) reporter constructs. Subsequently, these cells were further differentiated to cardiomyocytes. RNA was extracted and subjected to real-time RT-PCR and relative mRNA expression levels of both Luciferase and Puromycin in miPSCs (**A**) and cardiomyocytes (**B**) were determined. Expression level in pMSCV- GAPDHint2 transfected cells was determined as 1. Values are mean ± AVEDEV (n = 2) and normalized to Luciferase copy number.
(PDF)

S5 Fig IRF-8 mRNA expression in undifferentiated and differentiated miPSCs and MEF cells. RNA was extracted from the indicated cells and subjected to real-time RT-PCR. Relative mRNA expression levels of IRF-8 in miPSCs, cardiomyocytes, MEF, and RAW cells were determined. Expression level in RAW cells was determined as 1. Values are mean ± AVEDEV (n = 3).
(PDF)

S6 Fig H3K27me3 binding enrichment over the IRF-8 locus. Comparison of H3K27me3 occupancy over the IRF-8 3^rd^ intron (marked by red box) between two human cell types; Monocytes CD14+, IRF-8 permissive, and normal human lung fibroblasts (NHLF), IRF-8 restrictive. All experimental data are part of the ENCODE data set and were plotted with the UCSC genome browser (http://encodeproject.org/ENCODE/) [52, 53] that was made publicly available by the BROAD institute.
(PDF)

S1 Table Oligonucleotides used in this study. The primers used for real-time PCR were designed using PrimerExpress software (ABI) or previously described. For each primer, target organism is designated.
(PDF)
