# Supplementary figures and images for: The Third Intron of the Interferon Regulatory Factor-8 Is an Initiator of Repressed Chromatin Restricting Its Expression in Non-Immune Cells
Source: PLoS One. 2016 Jun 3;11(6):e0156812. doi: 10.1371/journal.pone.0156812 (PMC4892516; doi:10.1371/journal.pone.0156812)

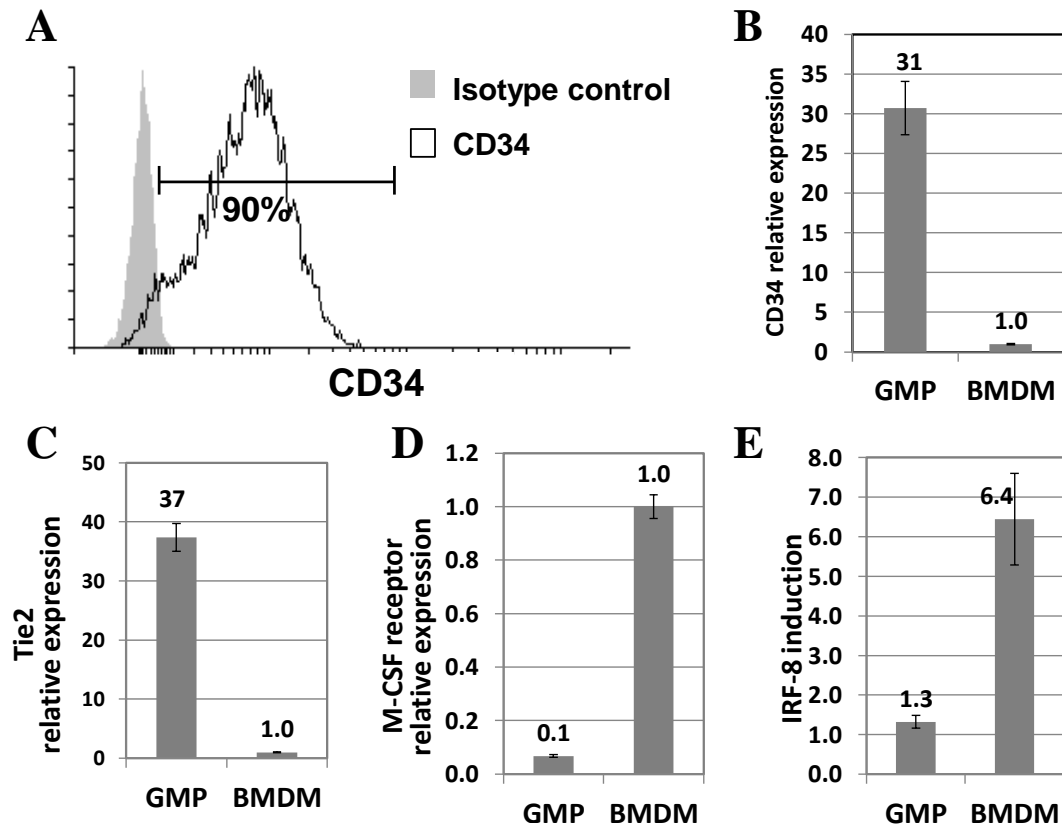

**S1 Fig. Characterization of primary mouse bone marrow derived GMP and BMDM cells.**

Supplement: S1 Fig — BM cells were harvested from the tibia and femur of 6–8 weeks old C57BL/6 mice, and cultivated with medium supplemented with IL-3 or M-CSF, resulting in GMP (CD34high) and BMDM cells, respectively. Cell characteristics were determined by analyzing GMP associated gene markers, CD34 and Tie2 [49, 50] AKA and macrophages associated marker, M-CSF receptor [51]. (A) Flow cytometry analysis of cell surface marker CD34 on GMP cells. qRT-PCR was employed to determine relative gene expression levels of CD34 (B), Tie2 (C) and M-CSF receptor (D). Expression level in BMDM cells was determined as 1. (E) Cells were treated with IFN-γ (100U/ml for 16 hrs. and IRF-8 induced expression in GMP and BMDM cells was calculated. IRF-8 expression level in untreated cells was determined as 1. Results shown are mean ± AVEDEV (n = 3). (PDF) [file pone.0156812.s001.pdf]

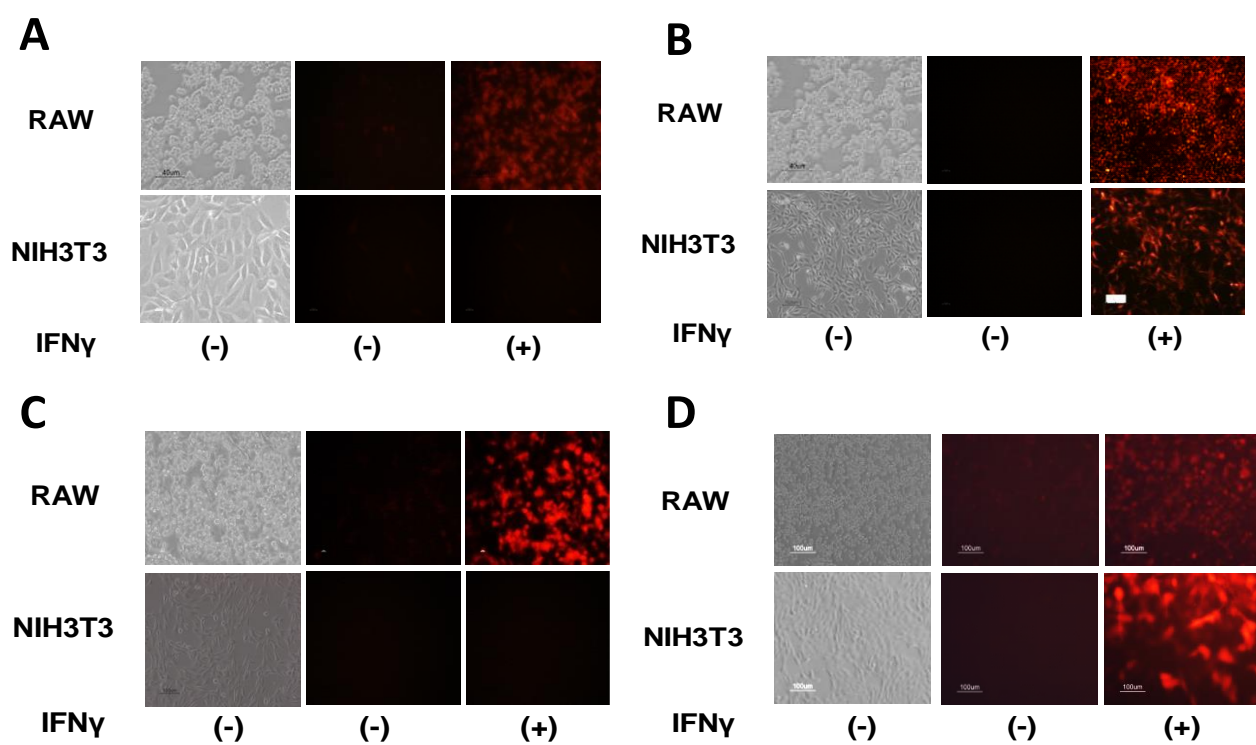

**Figure S2. Reporter gene expression in representative clones harboring BAC-IRF-8 constructs.**

Supplement: S2 Fig — RAW and NIH3T3 cells were transfected with the various BAC constructs and the fluorescence activity of the reporter gene in representative RAW and NIH3T3 stable clones, harboring 1–2 copies of the BAC reporter construct, was visualized under fluorescent microscope before and following 16 hrs of exposure treatment with IFN-γ (100 U/ml). Representative clones harboring BAC-IRF-8.1(A), BAC-IRF-8.2(B), BAC-IRF-8.3 (C) and BAC-IRF-8.4 (D) are shown. (PDF) [file pone.0156812.s002.pdf]

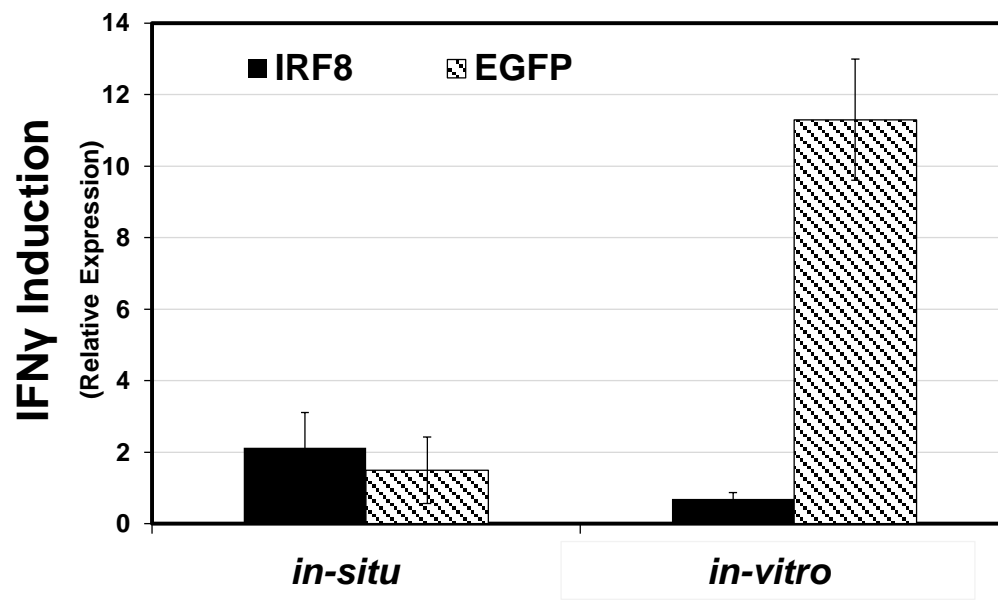

S3 Fig. mRNA expression levels of EGFP and IRF-8

Supplement: S3 Fig — NIH3T3 were transfected with BAC-IRF-8.1 VLoxP as described under Fig 6. To induce 3rd intron deletion within the cells (in-situ), stable clones were transduced with a retroviral vector encoding the VCre gene. For in-vitro deletion, the 3rd intron in BAC-IRF-8.1 VLoxP construct was initially deleted with the corresponding VCre recombinase in E. coli and subsequently transfected to NIH3T3 and stable clones were selected. The mRNA levels of the reporter gene (EGFP) and the endogenous IRF-8 were determined by real-time q-PCR from three independent clones for each deletion type; in-situ and in-vitro. Values are mean ± AVEDEV (n = 3). (PDF) [file pone.0156812.s003.pdf]

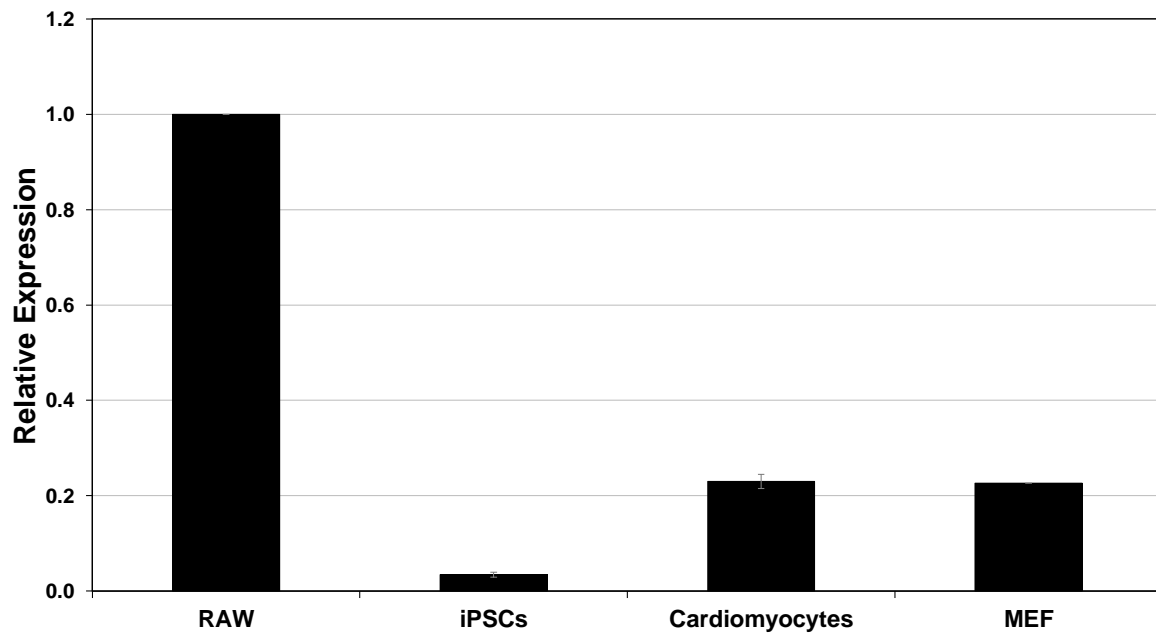

**S5 Fig. IRF-8 mRNA expression in undifferentiated and differentiated miPSCs and MEF cells.**

Supplement: S5 Fig — RNA was extracted from the indicated cells and subjected to real-time RT-PCR. Relative mRNA expression levels of IRF-8 in miPSCs, cardiomyocytes, MEF, and RAW cells were determined. Expression level in RAW cells was determined as 1. Values are mean ± AVEDEV (n = 3). (PDF) [file pone.0156812.s005.pdf]

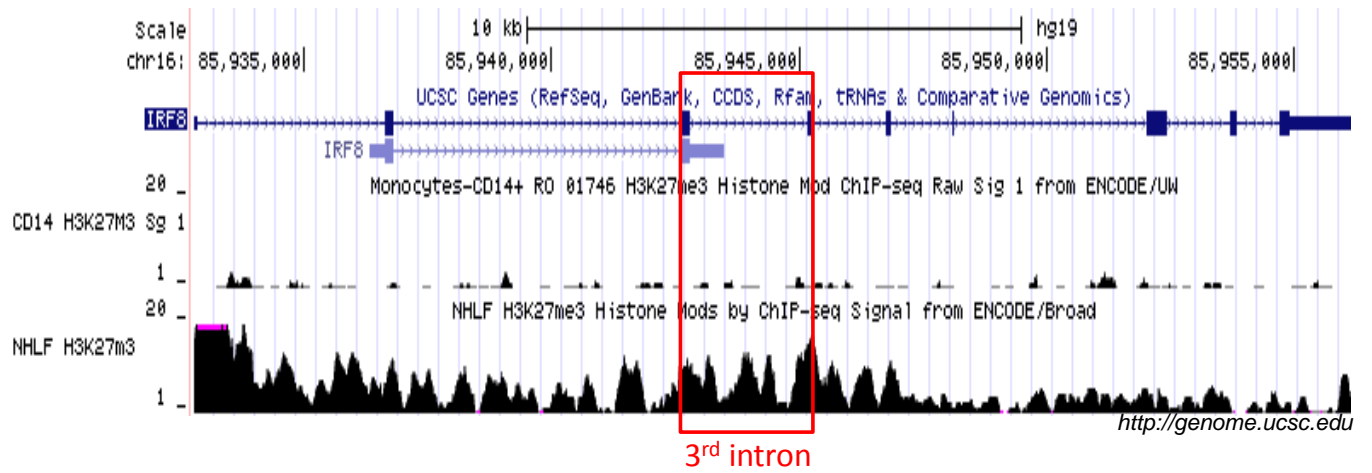

**S6 Fig. H3K27me3 binding enrichment over the IRF-8 locus.**

Supplement: S6 Fig — Comparison of H3K27me3 occupancy over the IRF-8 3rd intron (marked by red box) between two human cell types; Monocytes CD14+, IRF-8 permissive, and normal human lung fibroblasts (NHLF), IRF-8 restrictive. All experimental data are part of the ENCODE data set and were plotted with the UCSC genome browser (http://encodeproject.org/ENCODE/) [52, 53] that was made publicly available by the BROAD institute. (PDF) [file pone.0156812.s006.pdf]
