# Supplementary material for: The Third Intron of the Interferon Regulatory Factor-8 Is an Initiator of Repressed Chromatin Restricting Its Expression in Non-Immune Cells
Source: PLoS One. 2016 Jun 3;11(6):e0156812. doi: 10.1371/journal.pone.0156812 (PMC4892516; doi:10.1371/journal.pone.0156812)
Supplement: S4 Fig — miPSCs were transduced with either pMSCV-IRF8INT3 (IRF8Int3) or pMSCV- GAPDHint2 (GAPDHint2) reporter constructs. Subsequently, these cells were further differentiated to cardiomyocytes. RNA was extracted and subjected to real-time RT-PCR and relative mRNA expression levels of both Luciferase and Puromycin in miPSCs (A) and cardiomyocytes (B) were determined. Expression level in pMSCV- GAPDHint2 transfected cells was determined as 1. Values are mean ± AVEDEV (n = 2) and normalized to Luciferase copy number. (PDF) [file pone.0156812.s004.pdf]

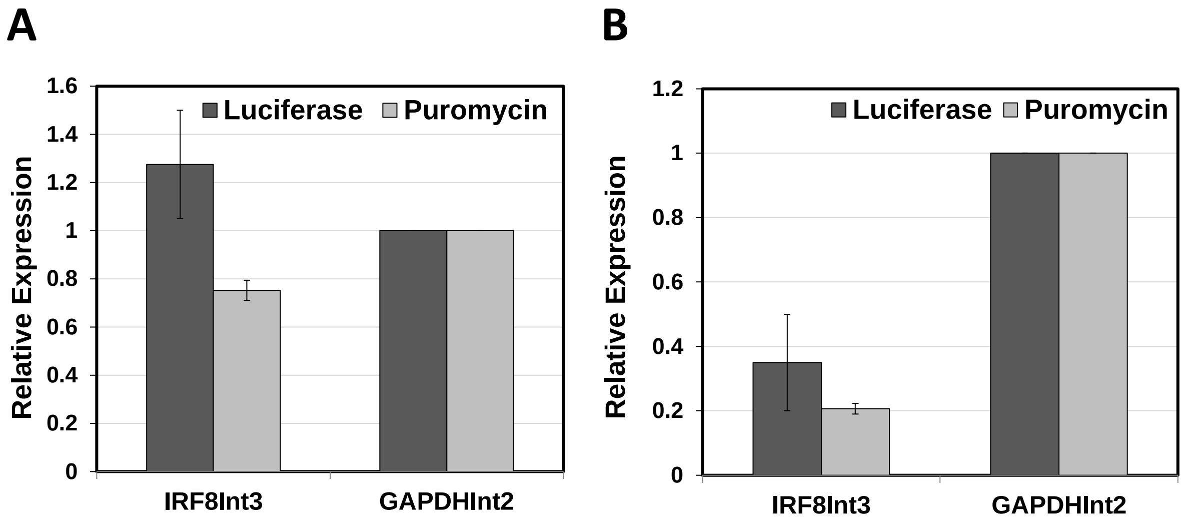

S4 Fig. Luciferase reporter gene and Puromycin relative mRNA expression in undifferentiated and differentiated miPSCs.
