# Supplementary material for: The Third Intron of the Interferon Regulatory Factor-8 Is an Initiator of Repressed Chromatin Restricting Its Expression in Non-Immune Cells
Source: PLoS One. 2016 Jun 3;11(6):e0156812. doi: 10.1371/journal.pone.0156812 (PMC4892516; doi:10.1371/journal.pone.0156812)
Supplement: S1 Table — The primers used for real-time PCR were designed using PrimerExpress software (ABI) or previously described. For each primer, target organism is designated. (PDF) [file pone.0156812.s007.pdf]

Table S1. Oligonucleotides used in this study.

**Name**		**Sequence (5' → 3')**
**mIRF-8 (cDNA)**	For	GGCAGTGGCTGATCGAACA
	Rev	GGTCTTCTCATCATTTTCCCAGA
**mIRF-8 3rd intron**	For	GCTTAACGCGTGTAACTATCTGTTGGGACC
	Rev	GCTATACGCGTCTATGGGAAAGGGGACAGAC
**mGAPDH (cDNA)**	For	AGGTCGGTGTGAACGGATTTG
	Rev	TGTAGACCATGTAGTTGAGGTCA
**mGAPDH 2nd intron**	For	GCTTAACGCGTGGATCCGGATGAGGTGGCCGAAGCGC
	Rev	GCTATACGCGTGGATCCACTCCTCATGGGTCTGTAGT
**mIRF-8 int3 amplicon1**	For	AGGTTACGCTGTGCTCTGAACA
	Rev	TCTAGCCCTGTGGAGACTGAGG
**mIRF-8 int3 amplicon2**	For	CCTCAGTCTCCACAGGGCTAGA
	Rev	AAGAGAGAACAACTCTGGTGAGCTAA
**mIRF-8 int3 amplicon3**	For	TTAGCTCACCAGAGTTGTTCTCTCTT
	Rev	AGTTTATGCTGAGCTCCGGG
**mIRF-8 int3 amplicon4**	For	CCCGGAGCTCAGCATAAACT
	Rev	AGGGAATCCTGCATCACAGACT
**mIRF-8 int3 amplicon5**	For	AGTCTGTGATGCAGGATTCCCT

	Rev	CACACCGAAGCCATCAGTGA
**mIRF-8 int3 amplicon6**	For	TGTGTCACTGCTGGAGAGGATAAC
	Rev	GCCAGTGTGCGTTCATTCC
**mIRF-8 int3 amplicon7**	For	ATGAACGCACACTGGCCTTC
	Rev	AGACAAAGGAGCCGGCCTT
**mIRF-8 int3 amplicon8**	For	CTCCTTTGTCTTCGCAGTGATTT
	Rev	AGTCAGGGTCATTAACCAGATCAAG
**mIRF-8 int3 amplicon9**	For	CTTGATCTGGTTAATGACCCTGACT
	Rev	AGAGAGGCAGGCAAACCACTC
**mIRF-8 int3 amplicon11**	For	GTCTCTCCACCTGGATGAAGC
	Rev	CCCTGGGTACATTTGCCTCAGGA
**mIRF-8 int3 amplicon12**	For	GTTATTGTTCCTGCTGTGTCTCATG
	Rev	TGCTGACCCACTTTGTACCTCTT
**mIRF-8 int3 amplicon13**	For	AAGAGGTACAAAGTGGGTCAGCA
	Rev	TGTATACGTGCCACATGGGG
**mIRF-8 int3 amplicon14**	For	CCCCATGTGGCACGTATACACA
	Rev	AAGGTAGGGAGTGCCAGGTAA
**mIRF-8 int3 amplicon15**	For	ATGCACACGTATAAAAGGGCAA
	Rev	GACAAGCGCCAGACTTTGG
**mIRF-8 int3 amplicon16**	For	CCAAAGTCTGGCGCTTGTC

	Rev	TCTCATGGCCGGGTCATCT
**mIRF-8 int3 amplicon17**	For	AGATGACCCGGCCATGAGA
	Rev	GGAGTAGTGAGCGTCCTTCGC
**mIRF-8 int3 amplicon18**	For	AGCAGCTGCTGGTCAAA
	Rev	AGGGCAGCCGAGCACACTGC
**mIRF-8 int3 amplicon19**	For	CAGTGTGCTCGGCTGCC
	Rev	TCCATCTCAGGAACTTCGCTC
**Del int3**	For	GCTGGAGGTGTTATGTGAC
	Rev	CATCTCAGGAACTTCGCTCATG
**mc-fms [M-CSF receptor]**	For	AGCACGAGAACATCGTCAACC
	Rev	TTCGCAGAAAGTTGAGCAGGT
**mEZH2**	For	AGCACAAGTCATCCCGTTAAAG
	Rev	AATTCTGTTGTAAGGGCGACC
**mTie2**	For	GATTTTGGATTGTCCCGAGGTCAAG
	Rev	CACCAATATCTGGGCAAATGATGG
**mCD34**	For	AAGGCTGGGTGAAGACCCTTA
	Rev	TGAATGGCCGTTTCTGGAAGT
**tGFP**	For	CACCCTCGTGACCACCCTGA
	Rev	AGGGTGTCGCCCTTCGAACT
**Puromycin 1**	For	TGACCGAGTACAAGCCCACG

	Rev	GTAGTCGGCGAACGCGGCGG
**Puromycin 2**	For	GTTCGCCGAGATCGGCCCGC
	Rev	CCAGGAGGCCTTCCATCTGT
**Puromycin 3**	For	TTCACCGTCACCGCCGACGT
	Rev	TCAGGCACCGGGCTTGCGGG
**Luciferase 1**	For	CGCTGGAGAGCAACTGCATA
	Rev	TCCACCTCGATATGTGCATCTG
**Luciferase 2**	For	GCCCGCGAACGACATTTATA
	Rev	TTGGAAACGAACACCACGGT
**Luciferase 3**	For	GGGATTTCAGTCGATGTACACG
	Rev	TCCCTATCGAAGGACTCTGGC
